# Lifestyle interventions on Alzheimer's disease and related dementias: Social and structural determinants of implementation effectiveness and sustainability

**DOI:** 10.1002/alz.71957

**Published:** 2026-11-10

**Authors:** Ganga S. Bey, Sheina Emrani, Erin R. Kulick, Cristina A. F. Román, Ileana De Anda‐Duran, Miriam T. Ashford, Rory Boyle, Omonigho Michael Bubu, Jasmine Dixon, Sanne Franzen, Pei‐Yang Gao, Kristen George, M. Aaron Guest, Margo B. Heston, Patricia Heyn, Daija Jackson, Sudarshan Krishnamurthy, Jeffrey S. Phillips, Awaale F. Rirash, Jayalakshmi Viswanathan, Preeti Pushpalata Zanwar, Megan Zuelsdorff, Shana D. Stites

**Affiliations:** ^1^ Department of Epidemiology, Gillings School of Global Public Health University of North Carolina at Chapel Hill Chapel Hill North Carolina USA; ^2^ Perelman School of Medicine University of Pennsylvania Philadelphia Pennsylvania USA; ^3^ Department of Epidemiology and Biostatistics Temple University College of Public Health Philadelphia Pennsylvania USA; ^4^ Keck School of Medicine University of Southern California Los Angeles California USA; ^5^ Kessler Foundation West Orange New Jersey USA; ^6^ Department of Epidemiology, Celia Scott Weatherhead School of Public Health and Tropical Medicine Tulane University New Orleans Louisiana USA; ^7^ Department of Psychiatry and Behavioral Sciences University of California San Francisco San Francisco California USA; ^8^ Penn Frontotemporal Degeneration Center Department of Neurology, Perelman School of Medicine, University of Pennsylvania Philadelphia Pennsylvania USA; ^9^ Departments of Psychiatry, Neurology, and Population Health, NYU Grossman School of Medicine New York University New York New York USA; ^10^ Department of Neurology Brigham and Women's Hospital Boston Massachusetts USA; ^11^ Department of Neurology and Alzheimer Center Erasmus MC University Medical Center Rotterdam the Netherlands; ^12^ Department of Neurology and Innovation Center for Neurological Disorders Xuanwu Hospital Capital Medical University Beijing China; ^13^ Department of Public Health Sciences University of California Davis Davis California USA; ^14^ Center for Innovation in Healthy and Resilient Aging Arizona State University Phoenix Arizona USA; ^15^ Edward and Pearl Fein Memory and Aging Center, Department of Neurology Weill Institute for Neurosciences University of California San Francisco San Francisco California USA; ^16^ Virginia Geriatric Education Center, Virginia Center on Aging Virginia Commonwealth University Richmond Virginia USA; ^17^ School of Public Health George Mason University Fairfax Virginia USA; ^18^ University of Colorado Anschutz Medical Campus Aurora Colorado USA; ^19^ Department of Behavioral Health Brooke Army Medical Center San Antonio Texas USA; ^20^ Department of Internal Medicine Wake Forest University School of Medicine Winston‐Salem North Carolina USA; ^21^ Department of Medical and Molecular Genetics Indiana University School of Medicine Indianapolis Indiana USA; ^22^ Stark Neurosciences Research Institute Indiana University School of Medicine Indianapolis Indiana USA; ^23^ Department of Pharmaceutical Sciences Irma Lerma Rangel College of Pharmacy Texas A&M University Health Science Center Kingsville Texas USA; ^24^ Hopkins Economics of Alzheimer's Disease and Services Center Johns Hopkins Bloomberg School of Public Health Baltimore Maryland USA; ^25^ School of Nursing University of Wisconsin–Madison Madison Wisconsin USA; ^26^ Wisconsin Alzheimer's Disease Research Center University of Wisconsin School of Medicine and Public Health Madison Wisconsin USA; ^27^ Perelman School of Medicine University of Pennsylvania Philadelphia Pennsylvania USA

**Keywords:** Alzheimer's disease and related dementias, disparities, health equity, implementation, intervention design, social determinants of health, structural determinants of health

## Abstract

Alzheimer's disease and related dementias (ADRD) pose a global public health challenge, and epidemiological evidence underscores profound disparities in ADRD risk and outcomes that are rooted in social/structural determinants of health (S/SDOH). Yet, these determinants are seldom integrated into intervention design or implementation strategies. In this review, we identify S/SDOH that influence the equitable implementation of evidence‐based ADRD lifestyle interventions across diverse groups and explore implications. Guided by Bronfenbrenner's ecological model, we: (1) examined theoretical foundations, focusing on how macrosystem and microsystem factors modulate implementation effectiveness across multiple intervention modalities; (2) synthesized empirical literature, highlighting successful and suboptimal intervention outcomes in marginalized populations; (3) identified critical gaps, especially where evidence on implementation across socially defined groups is lacking; and (4) discussed implications for enhancing design and policy for equitable ADRD intervention delivery. Our goal is to produce a conceptual and empirical synthesis that offers actionable guidance for clinicians, researchers, and policy makers.

## Highlights

- We examined social/structural determinants of health (S/SDOH) shaping Alzheimer's disease and related dementias (ADRD) intervention equity.
- Using an ecological framework, we discuss multilevel influences on intervention effectiveness and sustainability.
- To illustrate mechanisms, we used four evidence‐based lifestyle intervention targets: diet, physical activity, cognitive and social engagement, and health monitoring.
- This review highlights S/SDOH‐driven disparities in intervention access and effectiveness.
- We provide actionable guidance for facilitating equity‐focused ADRD interventions.

## 1 INTRODUCTION

Alzheimer's disease and related dementias (ADRD) pose a growing public health challenge worldwide, with incidence projected to rise sharply in the coming decades.^1^ Recent epidemiological evidence underscores profound disparities in ADRD risk and outcomes that are rooted in social and structural determinants of health (S/SDOH)—including, but not limited to, systemic inequities in socioeconomic resources, access to health care, and environmental exposures. Even still, these determinants are seldom integrated into intervention design or implementation strategies.^2^

Despite this growing recognition of the importance of S/SDOH, most ADRD lifestyle interventions are designed around isolating the exact, active ingredients that trigger behavior change. This approach is thought to enhance intervention effectiveness across different settings. Yet, integration of broader contextual factors is essential as these contexts shape individuals’ capacity to engage with and benefit from such interventions. Furthermore, implementation strategies frequently overlook systemic barriers (e.g., transportation inaccessibility, lack of health‐care coverage, or distrust in medical institutions rooted in historical and ongoing discrimination).^3^, ^4^ These oversights contribute to inequities in both access and outcomes, with ethnoracially and socioeconomically marginalized groups underrepresented in clinical trials and underserved in real‐world dissemination.^5^, ^6^ To enhance the impact and reach of ADRD interventions, it is imperative to design and implement strategies that are responsive to the lived realities and constraints shaped by S/SDOH.

Drawing on prior work,^7^, ^8^ the International Society to Advance Alzheimer's Research and Treatment (ISTAART) S/SDOH workgroup used Bronfenbrenner's Ecological Systems Theory^9^ as a conceptual model to elucidate how S/SDOH shape ADRD risk across contextual layers.^2^ This narrative analysis focused on the macrosystem—the structural and cultural forces that drive societal inequities and the microsystem—immediate, interpersonal environments that mediate individual experience, and concluded with a call to align intervention strategies with these multilevel social determinants to enhance equity in brain health outcomes.

In the current paper, we build upon that foundation by advancing a framework for achieving the national goals outlined in Healthy People 2030 of reducing the prevalence of dementia and improving brain health outcomes.^10^ Our approach is driven by a central question: How can implementation of evidence‐based ADRD lifestyle interventions be designed to maximize availability, uptake, adherence, effectiveness, and sustainability across diverse populations in the United States? Guided by Ecological Systems Theory, we synthesize the literature examining the roles that macrosystem‐ and microsystem‐level S/SDOH play in the equitable implementation of lifestyle interventions. This review gives particular attention to strategies that may be overlooked as critical to implementation relevance and sustainability within marginalized populations and concludes with targeted recommendations based on our findings. Our objective is to produce a conceptual and empirical synthesis that offers actionable guidance for clinicians, researchers, and policy makers, advancing ADRD intervention strategies that are effective and equitably implemented in real‐world settings.

We selected four ADRD‐related health behaviors that have been shown to be modifiable using lifestyle interventions—physical activity, diet, cognitive and social engagement, and health monitoring—to serve as examples of how multilevel S/SDOH contribute to variation in intervention implementation effectiveness across populations. The selection of these four intervention targets reflects strong evidence of their efficacy in ADRD prevention from randomized prevention trials that have been conducted cross‐nationally, including the U.S. Study to Protect Brain Health Through Lifestyle Intervention to Reduce Risk, or the POINTER study.^11^, ^12^, ^13^ The POINTER study, a 2‐year, phase III randomized controlled trial, adapted the multidomain lifestyle intervention paradigm, comprising physical exercise, nutritional counseling, cognitive and social stimulation, and cardiovascular health monitoring, from its Finnish predecessor, the FINGER trial, to a diverse American cohort.^11^, ^12^, ^14^, ^15^

Although ADRD prevention is shaped by exposures and health behaviors across the life span, a disproportionate focus is placed on midlife risk factors (ages 45–65) because the neuropathological changes of dementia begin to develop 10 to 20 years before any clinical symptoms appear. Intervening during this window offers the best opportunity to alter the disease's course; thus, prevention interventions are often designed to target the health behaviors of middle‐aged adults.^11^, ^12^, ^14^, ^15^ Mid‐life health behavior changes have been linked to prevention of ADRD and a host of other chronic diseases.^16^ To ensure the most accurate assessment of these mechanisms, the literature reviewed herein therefore includes studies that examine the relationship of S/SDOH with interventions on physical activity, diet, cognitive and social engagement, and health monitoring even if study outcomes were not ADRD specific.

To enhance the relevance of this review for advancing brain health equity specifically, for each macrosystem (Section 2) and microsystem (Section 3) determinant we: (1) examine theoretical perspectives on how the determinant influences the implementation effectiveness of lifestyle interventions targeting physical activity, diet, cognitive and social engagement, and health monitoring across diverse populations; (2) synthesize the empirical literature, highlighting both successful and suboptimal intervention outcomes on ADRD in marginalized populations; (3) identify critical gaps, particularly where evidence on implementation effectiveness specifically in relation to ADRD in marginalized populations remains limited; and (4) offer evidence‐based recommendations for strengthening policy, intervention design, and research to support equitable ADRD intervention delivery and sustainability in real‐world settings.

In alignment with our prior paper, this review will focus on select determinants at the macrosystem and microsystem level as they operate in the United States (Figure 1).^2^ We focus on the United States as a geographically and demographically diverse nation of robust systems and structures that are closely tied to the country's specific sociocultural and sociopolitical context and uniquely shape the implementation and sustainability of ADRD lifestyle interventions within that context. To connect isolated national observations to larger systemic patterns, we note broader global patterns where appropriate.

**FIGURE 1:**
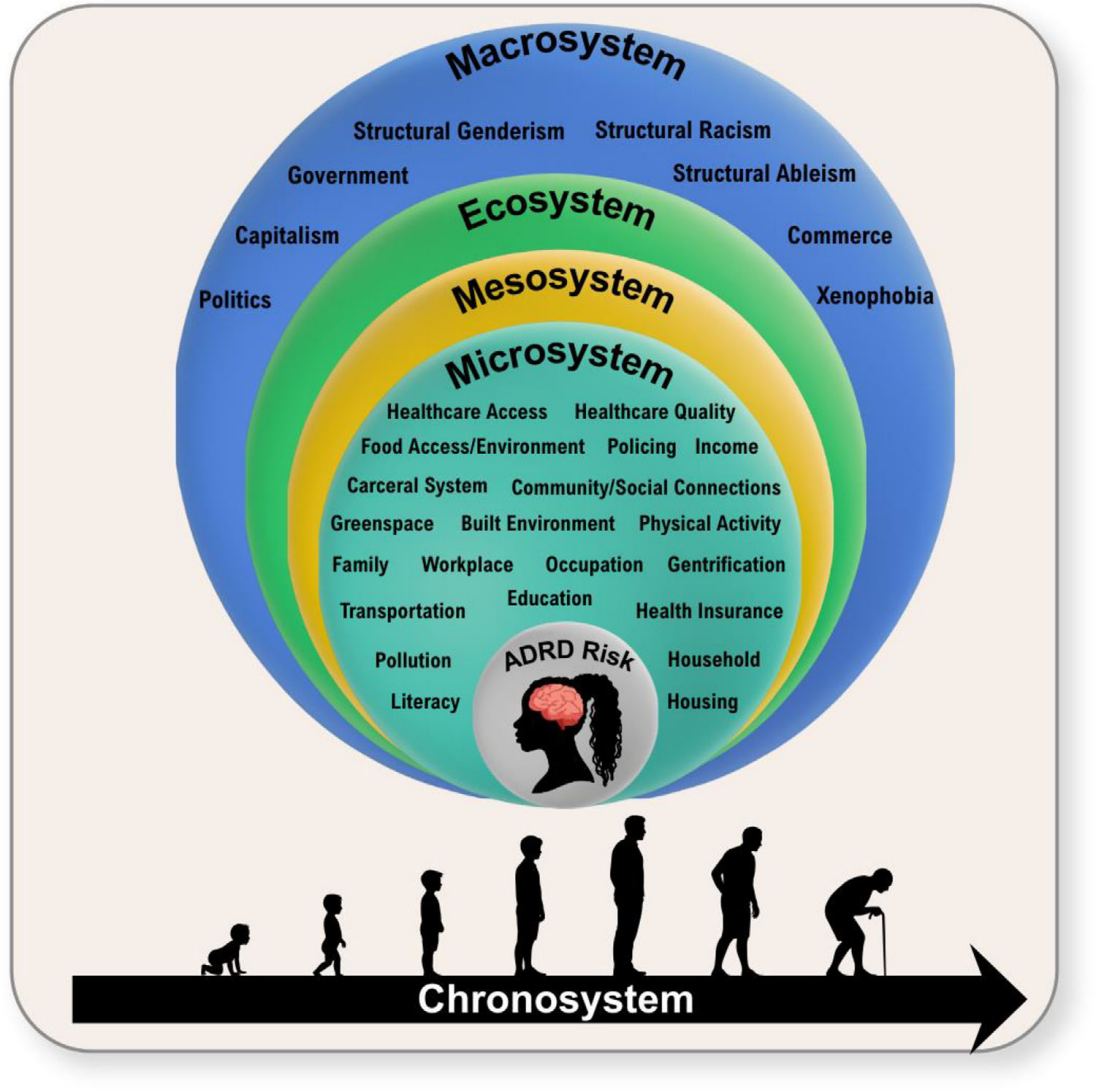
Macrosystem and microsystem structural and social determinants of health proposed to influence the relevance, effectiveness, and sustainability of lifestyle interventions on Alzheimer's disease and related dementias across diverse populations, adapted from Bronfenbrenner's Ecological Systems Theory. ADRD, Alzheimer's disease and related dementias

## 2 MACROSYSTEM‐LEVEL DETERMINANTS OF ADRD INTERVENTION IMPLEMENTATION EFFECTIVENESS

The US macrosystem encompasses the overarching cultural, economic, and political forces that shape the environments in which individuals live. These distal factors influence the conditions under which ADRD prevention interventions are implemented, accessed, and sustained. Here, we focus on several intersecting dimensions of the macrosystem within the United States that directly influence the real‐world application and effectiveness of evidence‐based ADRD lifestyle interventions: capitalism; structural racism, genderism, and ableism; and ethnocentrism. Throughout the paper, we emphasize how these macrosystemic forces are not abstract but intimately shape and are shaped by micro‐level experiences, behaviors, and outcomes within families, communities, and clinical settings. We show that addressing these macro‐level forces requires not only inclusive recruitment but also a critical evaluation of the content, delivery modes, and structural supports embedded in lifestyle intervention design. Table 1 provides a summary of the findings from the literature, key gaps we identified, and the specific recommendations described in the following sections.

**TABLE 1 alz71957-tbl-0001:** Example studies demonstrating mechanisms for the effects of macrosystem and microsystem factors on lifestyle interventions on Alzheimer's disease and related dementias.

Macrosystem		
S/SDOH	Quick reference points & example studies	Selected references
**Capitalism and related economic determinants**	Quick reference points: Political infrastructure at national, state, and local levels directly affects health outcomes through multiple pathways including but not limited to education, social welfare programs, politics, and income inequality. Commercial determinants of health and corporate political activities often shift policy in favor of commercial rather than public health interests. Across unhealthy commodity industries, products increase risk for chronic diseases. Food governance and politics further influence the ability to sustain health‐promoting diets through cost, availability, and quality. To build safe and sustainable physical activity practices depends on the availability of urban planning, public safety, and community engagement. Examples: A qualitative study of a lifestyle intervention in Black older adults, participants noted that prescribed dietary changes felt culturally foreign and financially burdensome, leading to lower adherence. An umbrella review found commercial marketing of unhealthy commodities, including tobacco, alcohol, and ultra‐processed foods, increases depression and cardiometabolic disorders, established dementia risk factors that undermine equitable prevention‐intervention effectiveness. A study that reviewed redlining maps and food environments showed that current neighborhoods that fell within historically redlined areas had a greater likelihood of having unhealthy retail food, even if the areas were now had increased economic and racial privilege. Gaps/opportunities for future studies: Increase awareness within marginalized communities of the health benefits of interventions like cognitive training. Examination of regional and state political and economic investment, particularly in low‐resourced regions, and implementation effectiveness of health‐related interventions.	^17^, ^19^, ^20^, ^21^, ^22^, ^23^, ^24^, ^25^, ^30^, ^247^, ^248^
**Structural racism**	Quick reference points: Structural racism shapes how public health interventions are designed, delivered, and evaluated. Geographic manifestations of structural racism—neighborhood segregation and systemic disinvestment—further constrain engagement in interventions. These structural forces influence access to safe walking spaces and culturally appropriate healthy foods. Populations facing the digital divide are also impacted, especially when interventions rely on computer‐based training. “Positive” stereotypes (e.g., Minority Myth) can obscure disparities, restrict data collection, and perpetuate inequities. Examples: Participants living in historically redlined neighborhoods may encounter high traffic, poor sidewalk conditions, or concerns about safety that limit their ability to complete physical activity components of a structured intervention. Pharmacists prescribed anti‐hypertensive medication placed in Black‐owned barbershops, reducing systolic blood pressure compared to controls, with significant pressure drops lasting over a year, demonstrating its sustainability when coupled with community‐engagement. Gaps/opportunities for future studies: Evaluation of content, delivery modes, and structural supports embedded in intervention designs and its effect on implementation effectiveness and adherence. Examination of sustainability of interventions across racial groups and incorporation of modification to amplify effectiveness.	^30^, ^31^, ^249^
**Structural genderism**	Quick reference points: Operating alongside sexism, transphobia, and homophobia, structural genderism creates compounded barriers for women, LGBTQ+ individuals, and gender‐diverse populations in the implementation and effectiveness of ADRD interventions.	^47^, ^49^, ^50^, ^250^, ^251^
	Sexism, as a foundational element of structural genderism, contributes to significant disparities in caregiving burden, economic security, and health system engagement. Structural genderism also operates through the erasure or marginalization of LGBTQ+ populations in aging and dementia research through discrimination, misgendering, and systemic exclusion from clinical settings, and undermining trust, deterring participation in prevention‐focused trials. Quick reference points: Digital interventions such as home‐based cognitive training may also inadvertently marginalize gender‐diverse individuals through data privacy risks or the absence of inclusive design. Biases are further compounded by policy‐level failures, including the lack of LGBTQ+ competency requirements in clinical trial protocols, or limited protections against discrimination in long‐term care and insurance coverage. Examples: An analysis of a community‐based cognitive wellness program for older adults in California found that lesbian and gay participants were less likely to attend follow‐up sessions when group facilitators used heteronormative language or failed to acknowledge the loss of same‐sex partners. A qualitative study of LBGTQ+ older adults in community‐based health programs, participants reported feeling hyper‐visible or tokenized in group settings, with facilitators often unprepared to navigate issues related to identity disclosure, partner loss, or medical mistrust. Gaps/opportunities for future studies: Institutional actions that incorporate inclusive designs welcoming gender‐diverse individuals in research agendas, service models, and health‐promoting interventions. Studies examining factors like support/compensation for women who are caregivers, expansion of study partners beyond the nuclear family, alleviation of data privacy risk, and the incorporation of inclusive‐based practices on implementation effectiveness of ADRD health‐related interventions.	
Structural ableism	Quick reference points: Structural ableism privileges certain physical, sensory, and cognitive capacities, implicitly defining health, participation, and success in ADRD interventions. Many intervention protocols exclude individuals with visual, hearing, mobility, or cognitive impairments, with individuals with intellectual and developmental disabilities, who face significantly elevated risk for early‐onset dementia, particularly vulnerable to this pattern of exclusion. Access to many exercise facilities and ability to prepare food remain physically inaccessible to individuals with mobility impairments, chronic pain, cardiorespiratory limitations, fine motor impairments, visual disabilities, or fatigue‐related conditions. Transportation barriers complicate adherence to exercise, intervention appointments, and nutritional changes for individuals with disabilities. Digital cognitive platforms, peer‐group formats, or health coaching may lack design elements that support participants with sensory impairments, learning disabilities, or neurodivergence. Examples: A review of 97 dementia‐related interventional study protocols found that 86% excluded individuals with cognitive disabilities, and more than half excluded participants with visual impairments. In qualitative studies with individuals with Down syndrome, the “impact of the lack of shared diagnosis” was identified as a theme. It was noted that without information about ADRD being sensitively and appropriately shared with individuals with Down syndrome, they cannot be positioned as authorities over their ADRD and related interventions. Gaps/0pportunities for future studies: Expand eligibility criteria and incorporate universal design principles in digital and in‐person formats and include anti‐ableism frameworks into research, policy, and funding structures. Measure accessibility and participation among disabled participants. Incorporate transportation and accessible features for health interventions like exercise (e.g., training in inclusive fitness, adaptive equipment), cooking (compensatory strategies for fine motor and visual impairments), and cognitive training (e.g., alternatives for iPad‐based delivery of training for visual, hearing, or mobility impairments).	^53^, ^54^, ^55^, ^59^, ^60^, ^61^, ^252^, ^253^, ^254^
**Xenophobia**	Quick reference points: Xenophobia shapes how individuals perceive, access, and engage with ADRD interventions. Language, religious beliefs, and perceived immigration status or cultural background interact with systems to promote or discourage involvement in ADRD interventions. Frequently, dementia prevention trials exclude participants who are not English proficient. Cultural and religious guided diets are not often considered and can conflict with prescription lifestyle changes. Few interventions have directly measured or mitigated the effects of xenophobia or anti‐immigrant sentiment on implementation effectiveness and intervention trials often fall short of comprehensive cultural adaptation. Examples: A survey of Latine and Asian immigrants showed enforcement levels were associated to greater barriers to care, including delaying care, which inadvertently impacts participation in in‐person or community components of ADRD interventions. The ¡Viva Bien! Lifestyle Intervention adapted elements of the Diabetes Prevention Program to fit the cultural and linguistic needs of Latinas, resulting in improved adherence and engagement. Faith‐based health promotion programs in African American/Black communities have reported success in promoting physical activity and dietary changes through culturally aligned messaging and church‐based infrastructure. Gaps/opportunities for future studies: Expand studies examining faith‐based health promotion for various religions and its effect on implementation effectiveness of ADRD interventions. Promote culturally and linguistically appropriate materials, trust‑building strategies (e.g., partnerships with community and faith‑based organizations), and legal‑services referrals. Advocate for policy reforms (e.g., health policies) that ensure undocumented or refugee participants can access prevention programs without fear. Acknowledge and consider the effect of xenophobia or anti‐immigrant sentiment on recruitment, retention, and implementation effectiveness at both regional and national levels.	^5^, ^64^, ^65^, ^66^, ^70^, ^255^, ^256^, ^257^
**Neighborhood Nutrition, food access, & food environment**	Quick reference points: The capacity of individuals to adopt and sustain healthy diets is shaped by constraints within their immediate environment. Food insecurity is a major barrier to implementing lifestyle modification interventions. Disparities underscore that promoting adherence to specific diets alone without addressing food availability and affordability may limit the effectiveness of health–behavior interventions, including cognitive and physical ones, and even exacerbate inequities. Several food access programs may strengthen or complement diet interventions in ADRD prevention (e.g., food preparation and delivery assistance). Community‐tailored approaches, including culturally adapted mobile markets and partnerships with food banks, further improve the feasibility and sustainability of dementia‐prevention strategies. Examples: Older adults who participate in SNAP have shown slower cognitive decline and better nutritional status, likely through improved access to a wider range of foods. Food environments separate from food insecurity impact dementia risk. A study of > 6000 community‐dwelling adults from the Health Care and Nutrition Study showed that individuals age 50+ who resided in neighborhoods characterized by low‐income, low‐vehicle access, and low‐fat milk prices had a higher risk of dementia. Qualitative data pointed to specific food environment factors linked to healthier food purchasing and consumption. Gaps/opportunities for future studies: Integration of food access in multidomain ADRD interventions. Effect on health‐care use and caregiving responsibilities on adherence and implementation effectiveness for nutrition‐based ADRD interventions.	^19^, ^80^, ^81^, ^88^, ^92^, ^94^, ^257^, ^258^, ^259^, ^260^
**Pollution**	Quick reference points: Strong evidence shows that environmental pollutants detrimentally affect health, including increased risk for ADRD. Research suggests noise and light pollution may be associated with ADRD through mechanisms such as sleep disruption, chronic stress, and cardiometabolic disease. Food, soil, and water contamination (e.g., pesticides, heavy metals, endocrine‐disrupting chemicals, plastic packaging that degrades upon heating) may blunt the impact of interventions, particularly those related to nutrition. Less is known about the impact of light and noise pollution on ADRD interventions, but they are likely to have indirect effects via factors like poor sleep. Example: A study showed that greater air pollution discourages physical activity and outdoor leisure time, particularly among women. Mixed results regarding whether aerobic intake in high air pollution is related to higher rates of cardiometabolic disease, which can exclude participants from intervention trials. Gaps/opportunities for future studies: Explicit studies on pollution's role in moderating participants’ adherence to and benefit from lifestyle interventions to prevent ADRD, especially among populations in high pollution settings. Test strategies to mitigate pollution effects such as facilitating participants’ air quality monitoring, identification of indoor exercise sites, training to avoid foodborne contaminants,^96^ and providing tools to reduce nighttime light and noise exposure.	^96^, ^97^, ^99^, ^100^, ^106^, ^110^, ^114^
**Greenspace & built environment**	Quick reference points: A large‐scale Census study showed Medicare beneficiaries that had more access to “greenness” was associated with a lower risk of ADRD. Greenspace functions as a supportive microsystem that may amplify the effectiveness of intervention activities by reducing competing psychological and physiological burdens. Lower income communities and ethnoracially minoritized neighborhoods consistently have significantly less greenspace, fewer street trees, and lower quality natural environments. Assumptions of universal access to greenspace may inadvertently reduce equitable participation and adherence. Even when individuals are highly motivated and interventions are well designed, environmental constraints may limit the feasibility or potential impact of behavior‐change strategies. Examples: There is evidence that natural environments reduce physiological stress markers, improve mood, enhance attentional capacity, and lower allostatic load. Gaps/opportunities for future studies: Integrating built environment considerations into ADRD intervention planning (e.g., indoor or virtual alternatives) may help achieve equitable implementation. Examine the effects of greenspace on other ADRD‐related interventions, including cognitive training, nutritional interventions.	^130^, ^131^, ^134^, ^261^, ^262^, ^263^
**Transportation**	Quick reference points: Regular attendance at structured interventions depends on access to safe, affordable, and frequent transportation. Transportation barriers can substantially reduce participation and retention across dementia prevention interventions, particularly in minoritized and rural populations. The ability to improve dietary habits can hinge on being able to access certain foods via reliable and safe modes of transportation. Physical activity and health monitoring interventions are especially affected when participants must travel to centralized locations. Transportation‐related implementation outcomes are rarely reported in a standardized way, which needs to be improved upon.	^41^, ^80^, ^135^, ^136^, ^137^, ^138^, ^141^, ^144^, ^146^, ^147^, ^264^
	Examples: In a study of 50 Black Americans who participated in focus groups around participating in aging and clinical research, they indicated that travel vouchers or a transportation provision would decrease barriers to participation. A study of urban‐ and rural‐dwelling older adults showed transportation to be a primary concern for rural‐dwelling older adults when participating in research, with participants indicating they would not have participated in ADRC research without being provided with transportation. Within urban groups, transportation was discussed but was not identified as a deciding factor. In a study of Latinx adults, older adults, and caregivers, focus groups revealed the importance of moving beyond traditional monetary benefits (i.e., participant payment) to include things like transportation expenses, lost hourly labor, and caregiver fees/duties. Gaps/opportunities for future research: Reporting transportation‐related implementation outcomes. Examination of implementation effectiveness using online formats with support for digital access and literacy. Home‐based versus in‐person visits (with vouchers, support for caregivers, etc.) on implementation effectiveness across the ADRD interventions.	
**Housing**	Quick reference points: Housing precariousness, or housing adequacy, can impact a person's likelihood of being recruited into clinical trials and engagement in targeted population‐level interventions, as housing precariousness–related health conditions, as well as housing stability itself, may represent exclusion criteria. Housing precariousness can also impact the effectiveness of ADRD interventions by impairing participants’ adherence, due to challenges related to housing affordability, quality, and security/stability. Individuals experiencing high housing cost burdens may have fewer financial resources available for other essential needs, limiting their ability to adhere to diet‐based interventions, maintain gym memberships, or participate in other interventions that entail financial costs, as well as restrict individuals’ ability to prepare healthy meals, further undermining adherence to diet‐focused intervention components. Living in crowded or noisy housing environments, poor quality housing, or housing in unsafe or historically marginalized neighborhoods could impede cognitive training interventions. Examples: A review of SDOH in the deep south (Mississippi) highlighted the importance of improving health outcomes through socially driven interventions that address high‐level SDOH, such as housing insecurity. Results from a study using National Study of Midlife in the United States data showed that housing insecurity was associated with more chronic conditions and poorer self‐rated physical health, especially in midlife Black adults, which has implications for ADRD risk and ability to participate in midlife and later life ADRD interventions. Gaps/opportunities for future research: Overall, there has been a dearth of studies examining the intersection of housing precariousness and intervention effectiveness. Studies examining ways to reduce financial burdens that can impede recruitment and adherence to ADRD health‐related interventions and boost implementation effectiveness is warranted.	^151^, ^154^, ^265^, ^266^, ^267^, ^268^, ^269^, ^270^
**Poverty & income**	Quick reference points: Participants living in poverty may face competing financial and survival priorities that undermine their ability to engage consistently with interventions. A paradox is created, such that those who may benefit most from preventive interventions, due to heightened risk for ADRD from social disadvantage, are often those least able to engage with and sustain them. Community‐level poverty is a key driver of neighborhood barriers to health‐enabling infrastructure (e.g., safe walking environments, community centers, broadband internet access). Intervention designs that recognize that health behaviors are constrained by economic hardship will allow for better results and impact.	^271^, ^272^, ^273^, ^274^, ^275^
	Examples: A substantial number of intervention study models presume financial security and all that comes with it; however, these assumptions may not hold for individuals experiencing poverty or lower income levels. Studies have shown that financial hardship can lead to reduced executive function and decision‐making flexibility, which in turn may impair one's ability to adhere to cognitively demanding or time‐sensitive intervention protocols. Gaps/opportunities for future studies: Reporting and analyzing income as it relates to implementation effectiveness of ADRD‐related lifestyle interventions. Examination the effects of ADRD interventions that include mechanisms to offset the real‐time impacts of poverty, as providing transportation vouchers, child or elder care support, or culturally relevant food subsidies.	
**Community & social connections**		
**Policing & the carceral system**	Quick reference points: Although limited, research suggests that incarceration and aggressive or discriminatory policing practices contribute to chronic stress, trauma, and institutional mistrust that in turn impact cognitive decline and increased risk of ADRD. Policing and carceral factors disproportionately affect ethnoracially minoritized communities. Individuals on probation or parole may face restrictions that limit their ability to participate in ADRD interventions, especially those that require travel to a central location. Adults reentering society after incarceration often encounter many barriers (e.g., unstable housing, food insecurity) that make engagement in multicomponent interventions extremely difficult or nearly impossible without substantial systemic and structural supports. To promote equitable and sustainable participation, ADRD lifestyle interventions must integrate strategies tailored to reentry populations. Embedding cognitive health promotion within broader reentry support ecosystems, rather than requiring individuals to access siloed services independently, is a crucial step toward equity. Examples: A study on the cognitive trajectories of older adults who had previously been incarcerated (data from the Health and Retirement Study), showed that previously incarcerated older adults were more likely to demonstrate a cognitive trajectory related to lower cognition scores overall and steeper cognitive decline over time, which can result in exclusion of participation in such clinical trials. However, these relationships lessened or became statistically insignificant after accounting for sociodemographic, health, and education factors. A study of incarcerated individuals within the South Carolina Alzheimer's disease registry showed a higher prevalence of ADRD among incarcerated individuals with those incarcerated at earlier ages showing a higher rate of dementia at earlier ages. Gaps/opportunities for future studies: Investigation of how interactions with carceral systems impact implementation of ADRD‐related health interventions. Examination of the impact of flexible or decentralized delivery (e.g., mobile health units, virtual programming), partnering with reentry and community‐based organizations, embedding trauma‐informed practices, among other strategies, can improve implementation in this population.	^158^, ^159^, ^160^, ^161^, ^163^, ^276^
**Household & family**	Quick reference points: The integration of family/chosen family/caregivers in health promotion interventions has been shown to increase patient adherence and engagement in activities focused on improving physical health, cognitive function, and nutrition. Cultural norms around caregiving, familial roles, health beliefs, and stigma may influence family/chosen family/caregivers’ willingness or ability to engage in structured interventions. Conversely, interventions that rely heavily on household/family/chosen family/caregiver participation may reduce intervention effectiveness, especially in the context of other obligations such as employment or managing their own health concerns. Regardless, there are limited data on the relationship between household/family/chosen family/caregivers on multidomain ADRD interventions, especially in racially, ethnically, culturally, identity, and linguistically diverse groups.	^277^, ^278^, ^279^, ^280^, ^281^, ^282^, ^283^
	Examples: Research has shown that increased understanding of the benefits of physical activity and collaboration between caregivers and physical activity providers in implementing interventions was a key factor to facilitating physical activity in individuals with dementia. A study of family caregivers showed that active participation of the family caregiver in cognitive rehabilitation interventions enhanced treatment adherence and overall engagement among individuals with dementia. Other studies have suggested that support strategies from caregivers or households related to nutrition (e.g., meal preparation, eating together) provide special opportunities for nutrition and social engagement inside and outside of the home. Gaps/opportunities for future studies: Interrogation of household/chosen family/family participation in interventions for those in the preclinical or prodromal stages of ADRD. Examination of how cultural, socioeconomic, and geographic factors influence family/household member involvement in the implementation and effectiveness of ADRD‐focused interventions.	^277^, ^278^, ^279^, ^280^, ^281^, ^282^, ^283^
**Physical activity**	Quick reference points: ADRD studies and intervention trials have demonstrated the importance of exercise‐anchored dementia‐risk reduction, but they have also introduced barriers, such that the most effective approaches often require substantial resources that can constrain scalability, access, and inclusion. Macrosystem (e.g., cultural norms, legal/religious factors) and microsystem (e.g., neighborhood safety, transportation, personal health status [mobility, visual/hearing impairment], etc.) factors may influence people's ability to engage in or benefit from physical activity. Healthy People 2030 identifies five key S/SDOH domains that shape a person's ability to initiate and sustain activity and should be considered for future intervention studies. Implementation guides, structured exercise prescriptions, incorporating qualified community programs, and payer supports are all resources that can be used or considered for increasing physical activity within multidomain ADRD interventions. Examples: In older adults, neighborhood walkability, pedestrian safety, and access to greenspaces are consistently associated with higher activity and adherence, and in several reports, with better late‐life cognitive outcomes, whereas historically redlined neighborhoods show lower and less friendly neighborhood walking with higher social vulnerability. A systematic review and meta‐analysis of longitudinal studies showed that older adults who engaged in higher levels of physical activity have a reduced risk of cognitive decline, with an estimated 18% reduction of risk for dementia (as associated with physical activity). Gaps/opportunity for future studies: Few trials have examined the intersection of important resources and factors, including neighborhood safety, mobility, multimorbidity, cultural norms, transportation access, and other aspects, on physical activity implementation effectiveness. Examination of how built clinic‐to‐community networks, funding transport, and adaptive options can improve implementation effectiveness of these interventions.	^12^, ^96^, ^284^, ^285^, ^286^, ^287^, ^288^, ^289^, ^290^, ^291^, ^292^
**Social networks & isolation**	Quick reference points: Social support is both a motivational catalyst and an adherence mechanism, particularly in interventions requiring sustained behavioral change. The social component of health behavioral change programs, like cognitive stimulation, contributes to better adherence and motivation. Nutritional and physical interventions are also more effective when they incorporate social support, like with peer‐delivered or “buddy” interventions. Programs that embed opportunities for peer connection cultivate a sense of belonging that buffers against stress, isolation, and withdrawal and improves implementation effectiveness.	^172^, ^173^, ^174^, ^175^, ^176^, ^177^, ^178^, ^181^
	Examples: Studies have shown that group‐based physical activity interventions demonstrate stronger cognitive outcomes than solitary exercise. For example, older adults who exercised with others had a significantly lower risk of cognitive impairment. Cognitive stimulation therapies delivered in group formats show measurable improvements in memory, communication, and quality of life, and peer interactions have been shown to provide collaborative problem solving and discussion opportunities, each of which independently stimulates cognitive function. Studies show that older adults who receive encouragement from peers or family members are more likely to adopt and maintain healthy dietary habits, such as increased fruit and vegetable intake. Gaps/opportunity for future studies: Measure social isolation, loneliness, network size/quality, and perceived belonging to better understand what factors are most likely to improve ADRD intervention implementation effectiveness. Incorporate social network mapping and loneliness metrics into intervention trials and provide digital literacy resources to reduce technology‐driven isolation and inequities in remote program delivery.	
**Gentrification**	Quick reference points: Lower income, historically marginalized older adults experience the greatest consequences, such as displacement, loss of social cohesion and connection, food insecurity, poorer physical and mental well‐being, less access to age‐appropriate resources and recreation, and more chronic health difficulties, all of which can have negative downstream cognitive impacts. Though knowledge is limited, older adults living in gentrified areas experience potential barriers when trying to access or engage in interventions to maintain their cognitive health, resulting in a reduced impact from multidomain interventions. Sufficient infrastructure that may be lost through gentrification may lead to barriers to implementation of interventions. For example, displacement due to pricing out of neighborhoods can disrupt social and community networks and therefore impact access to intervention resources. Examples: In a study of older adults in gentrifying urban neighborhoods in New York City (USA), participants were more likely to experience food insecurity compared to those in moderate‐ to high‐income neighborhoods. Social cohesion and health food outlets were identified as potential factors for minimizing food insecurity in gentrifying neighborhoods. In a study of Black adults’ experiences of aging in place in a gentrifying urban environment, participants identified consequences of neighborhood change as it relates to neighborhood identity, cultural displacement, housing, and financial instability. Older adults felt displaced even when remaining in their same neighborhood or household. Though not directly discussed, the direct experiences of displacement and interruption of social cohesion can impact multidomain interventions for ADRD through multiple pathways. Gaps/opportunities for future studies: Empirical data showing how gentrification affects implementation effectiveness across ADRD‐related health interventions and the specific barriers that make implementation challenging. ADRD lifestyle interventions should partner with trusted community organizations, tenant groups, senior centers, and neighborhood coalitions to preserve access in rapidly changing neighborhoods. Asset‐based approaches that leverage existing community strengths may help sustain intervention engagement.	^182^, ^183^, ^184^, ^185^, ^186^, ^187^, ^188^, ^189^, ^190^, ^191^
**Workplace/ occupation**	Quick reference points: Workplace characteristics, like job complexity and schedule flexibility, can shape opportunities to maintain brain health and engage in structured interventions by imposing structural and psychosocial constraints that limit individuals’ ability to engage in health‐promoting behaviors. Caregivers’ occupational burden is often underrecognized and experience comparable barriers,^227^ including reduced engagement in preventive health behaviors,^228^ missed medical appointments,^229^ and compromised nutrition and physical activity. Workplace‐based interventions are among the most effective approaches for promoting exercise and nutritional behaviors among adults in the United States.	^198^, ^199^, ^200^, ^201^, ^203^, ^204^, ^293^
	Examples: Studies show that shift workers frequently exhibit poorer dietary and exercise patterns, reflecting the challenges of irregular schedules, limited recovery time, and fatigue that hinder participation in structured exercise or meal preparation. Studies of team‐ and individual‐based programs targeting nutrition, physical activity, and energy balance were conducted at participants’ workplaces and achieved strong engagement. A study of female health‐care workers that involved a workplace‐based intervention incorporating motivational counseling, free access to an onsite gym, and participation in risk reduction classes significantly increased weekly exercise frequency and improved weight management compared to controls. Gaps/opportunities for future studies: Investigate the degree to which social and cognitive interventions can be successfully implemented with certain occupations. Intervention designs should include flexible schedules, remote options, or brief intervention modules and, to the extent possible, working with participants to engage in workplace‐based interventions may promote sustainability in ADRD lifestyle interventions.	
**School literacy**	Quick reference points: The term literacy covers different aspects of functioning, including fundamental literacy, digital literacy, and health literacy. In ADRD prevention research, literacy is rarely measured/reported, and education is usually categorized broadly. Sources of bias include cognitive and screening tests that can result in greater frequency of misdiagnosis among low educated or low‐literate participants, questionnaires or food logs that may be difficult to fill out, or informational materials in written form that may be more difficult to understand. Web‐based cognitive training platforms pose challenges for those with limited digital or fundamental literacy Examples: In diabetes research, peer health coaches can promote diabetes control in culturally, educationally, and linguistically diverse patient groups, supporting benefits for ADRD‐related intervention effectiveness. An umbrella review of electronic health interventions reported that limited digital literacy reduces participation and adherence among older adults, highlighting the need for technical training and simplified platforms in intervention delivery for older adults with cognitive impairment. Gaps/opportunities for future studies: Determine whether other delivery modalities, like visual tools or peer navigation, can reduce literacy‐related barriers. Finally, food logs, questionnaires, and cognitive training tasks should be evaluated for literacy‐related burden and potential measurement bias. Report/measure literacy and quality of education (vs. years of education) in relation to implementation effectiveness of ADRD interventions. Report/measure literacy and quality of education (vs. years of education) in relation to implementation effectiveness of ADRD interventions.	^212^, ^213^, ^214^, ^294^
**Education**	Quick reference points: Extensive evidence underscores the protective influence of education against cognitive decline. Ongoing health education is recognized as a critical component within ADRD interventions. Education has been shown to also notably influence health–behavior intervention adherence by improving health literacy, enhancing self‐efficacy, and increasing resource accessibility. Higher educated individuals generally demonstrating greater engagement in preventive behaviors and cognitive training and therefore derive greater cognitive benefits, and culturally adapted or community‐based health education initiatives have successfully increased dementia‐preventive behaviors among diverse communities, groups, and individuals. Examples: Large‐scale meta‐analyses consistently identify low education attainment as a prominent modifiable risk factor, attributing ≈ 9.3% of global dementia cases to insufficient education. In a community‐based study, education attainment lessened the strength of the relationships between cerebrovascular disease burden (i.e., white matter hyperintensities) on memory and language in individuals who identified as non‐Hispanic White but not non‐Hispanic Black or Hispanic.	^98^, ^210^, ^295^, ^296^, ^297^, ^298^, ^299^
	Gaps/opportunities for future studies: Investigate how comprehensive questionnaires that extend beyond binned educational attainment (e.g., some high school/high school, etc.) better characterize the effects of education on ADRD lifestyle intervention effectiveness. Future research and policy initiatives must prioritize educational equity and culturally sensitive intervention strategies to enhance the effectiveness and sustainability of ADRD prevention efforts across diverse populations.	
**Health‐care access**	Quick reference points: As a multilayered micro‐level social determinant of health, health care, including Medicare and health insurance, directly and indirectly impacts interventions for ADRD, contributing to or mitigating health disparities. More than 90% of adults over the age of 65 receive a percentage of Medicare coverage, with reliance on Medicare alone increasing over time. Even with Medicare coverage, out‐of‐pocket costs still exist and create a burden for individuals, their families, and the community, something that may deter or interfere with intervention participation. Disparities in health‐care access and quality have been shown to exist for individuals from culturally and/or linguistically diverse, disadvantaged, and minoritized backgrounds. Individuals who experience barriers to health‐care access or poor‐quality health care are more likely to have comorbid conditions which may preclude or exclude them from engaging in interventions. Health monitoring requires substantial resources and access to health‐care professionals and settings. Multifaceted clinical intervention studies have the potential to bring health‐care access and quality health care to communities that have the greatest need, but this requires significant and consistent resources. Examples: Studies have shown that individuals with comorbid medical conditions are at higher risk for experiencing cognitive decline and conversion to ADRD at earlier ages compared to individuals without such conditions, thereby potentially leading to exclusion of the most at‐risk individuals or implementing interventions too late. Results from a study using Health and Retirement Study data showed that older adults who experienced lower quality health care had an increased risk of dementia, with health‐care discrimination and dissatisfaction being most related to dementia risk. Gaps/opportunities for future research: Identify feasible and more affordable ways to monitor health and provide consistent care, including in‐home care programs. Provide provider and community health education and resources with access to multidisciplinary providers, health education around ADRD can also be provided to providers. Address access to vision/hearing care (e.g., insurance coverage, language barriers) and propose policy solutions (subsidized devices, universal coverage, tele‐audiology). Suggest partnerships with mobile clinics and community health workers to provide screenings in underserved neighborhoods.	^215^, ^216^, ^217^, ^218^, ^222^, ^223^, ^300^, ^301^, ^302^, ^303^

Abbreviations: ADRD, Alzheimer's disease and related dementias; LGBTQ+, lesbian, gay, bisexual, transgender, queer, and more; SNAP, Supplemental Nutrition Assistance Program; S/SDOH, social and structural determinants of health.

### 2.1 Capitalism and related economic determinants

The economic contexts in which lifestyle interventions for preventing ADRD—including increased physical activity, healthier diet, greater cognitive and social engagement, and increased health monitoring—occur profoundly shape access, adherence, and long‐term sustainability. Globally, these contexts are structured by a variety of economic systems, including capitalist, non‐capitalist, and mixed economies, through which economic priorities and policy decisions determine access to resources that support healthy aging and dementia prevention. Given the US focus of this review, we describe the mechanisms by which the racialized US economic system—capitalism—shapes the implementation effectiveness of ADRD interventions.

Capitalism, an economic system fundamentally characterized by profit‐driven production and distribution of goods and services,^17^ plays a central role in the differential allocation of health‐promoting resources and public services across communities.^2^, ^18^, ^19^, ^20^ As has been established by decades of interdisciplinary literature,^2^ US capitalism is historically distinctive in that the development of the nation's economy has relied on racialized systems of enslavement, dispossession, exclusion, and labor exploitation that have shaped the distribution of wealth, political power, and material resources across racialized groups. By subjecting public health infrastructure, social welfare policies, and efforts to address the historical disenfranchisement and marginalization of minoritized populations to racialized market‐driven priorities, capitalism in the US determines which populations can access and sustain the behaviors targeted by ADRD prevention interventions. As discussed further in Section 3, health behaviors are heavily shaped by the immediate physical and social environments which are the microsystem manifestations of these economic priorities and policies.

The impact of economic priorities and policy decisions on the implementation effectiveness of lifestyle interventions for ADRD prevention is particularly evident through commercial determinants of health.^2^, ^20^ Commercial determinants of poor health—defined as “the strategies and approaches used by the private sector to promote products and choices that are detrimental to health”^20^—along with corporate political activities such as lobbying, often shift policy toward commercial rather than public health interests.^21^ This dynamic is evident across unhealthy commodity industries (UCIs), such as tobacco, alcohol, gambling, processed food, social media, and so on, whose products contribute to ADRD risk indirectly through associated conditions such as depression, diabetes, cardiovascular disease, and obesity.^22^, ^23^ By failing to adequately regulate the disproportionate targeting of these UCIs toward marginalized—particularly ethnoracially marginalized—communities, US capitalism creates population‐specific barriers to increasing and sustaining physical activity, accessing healthy foods, and maintaining social engagement over the life course.[Table alz71957-tbl-0001]

Food governance, for example, affects the ability to maintain health‐promoting diets through its influence on food cost, availability, and quality. In the US, access to healthy food options is shaped by zoning laws, redlining, and food insecurity, which are largely driven by broader corporate interests and disproportionately adversely impact ethnoracially marginalized communities.^24^ Similarly, opportunities for safe and sustainable physical activity depend on urban planning, neighborhood safety, and community infrastructure that supports engagement and mobility.^25^, ^26^ Regular participation in cognitively and socially stimulating activities depends on access to education resources, community institutions, and shared public spaces that are perceived as physically and emotionally safe.^27^, ^28^ However, the distribution and accessibility of these environments are often determined by private development priorities and market‐driven investment patterns that disproportionately disadvantage ethnoracially and socioeconomically marginalized communities. Consequently, US capitalism influences both the availability of ADRD‐preventive resources and the environmental conditions needed to sustain health‐promoting behaviors.

#### 2.1.1 Key knowledge gaps

Despite growing recognition of the commercial determinants of health, the influence of economic systems and priorities on ADRD intervention design and implementation remains substantially underexplored. Specifically, few studies have empirically assessed UCIs or corporate political activity in relation to ADRD lifestyle intervention uptake and sustainability across diverse communities.

#### 2.1.2 Recommendations

Policies that strengthen public health infrastructure, regulate UCIs, and expand access to education, affordable housing, and social welfare programs may reduce the adverse effects of capitalist systems on the implementation and effectiveness of ADRD prevention interventions.^18^, ^19^, ^21^ Lifestyle‐based ADRD interventions must account for the unequal distribution of health resources across communities by incorporating culturally and contextually tailored approaches, prioritizing affordability and accessibility, and addressing the economic policy‐driven barriers that limit long‐term adherence to health‐promoting behaviors. Because these barriers are particularly salient among ethnoracially and socioeconomically marginalized populations, interventions designed to address the consequences of racialized capitalism can facilitate more equitable implementation, as further specified in section 4.2.2. Future research should leverage political economy frameworks^29^ to empirically examine how US capitalism, and specifically commercial determinants of health, shape the design, implementation, accessibility, and effectiveness of ADRD prevention interventions.

### 2.2 Structural racism

Structural racism shapes how public health interventions are designed, delivered, and evaluated. Through laws, institutional policies, and cultural norms, structural racism systematically disadvantages racially and ethnically minoritized populations. In ADRD research, longstanding underrepresentation of racially minoritized groups has limited our understanding of etiological heterogeneity and the development of culturally relevant intervention strategies.^3^, ^30^

A central mechanism of structural racism is the legacy of exclusion from, and mistreatment in, biomedical research, which has fostered deep mistrust of clinical institutions among racially marginalized communities. This mistrust—rooted in numerous unethical research practices such as the US Public Health Service Untreated Syphilis Study at Tuskegee—reduces the enrollment, engagement, and retention among diverse populations.^31^ Additionally, “positive” stereotypes, such as the model minority myth often applied to Asian Americans, obscure disparities, restrict data collection, and perpetuate inequities.^32^, ^33^ Consequently, ADRD intervention evidence remains mainly skewed toward White, higher socioeconomic status (SES) populations, limiting generalizability and translational impact.

Structural racism also influences the context in which interventions are implemented. Dietary prescriptions, like the Mediterranean–Dietary Approaches to Stop Hypertension Intervention for Neurodegenerative Delay (MIND) diet, may conflict with cultural food traditions or be financially inaccessible or unavailable, reducing adherence. Without adaptations that incorporate culturally relevant food practices, dietary interventions risk marginalizing non‐Western eating patterns and reinforcing racial hierarchies in nutrition science. Geographic manifestations of structural racism—neighborhood segregation and systemic disinvestment—further constrain engagement. These structural forces influence access to safe walking spaces and culturally appropriate healthy foods, as previously noted. Similarly, the assumption of reliable home internet access for computer‐based cognitive training, or ownership of a computer, may not hold in communities facing the digital divide, disproportionately affecting Black and Hispanic households.^34^, ^35^, ^36^, ^37^ These mechanisms illustrate how structural racism shapes the implementation landscape for ADRD interventions, affecting everything from participation and engagement to perceived relevance and sustainability.

#### 2.2.1 Key knowledge gaps

Most existing lifestyle intervention evidence come from predominantly White, high SES populations, thus limiting generalizability of how structural racism affects intervention uptake, adherence, and sustainability across different minoritized communities. For example, examinations of residential segregation, neighborhood disinvestment, health‐care discrimination, or access to safe spaces for physical activity are limited or non‐existent in intervention research. There is also limited work on culturally incongruent intervention content, including dietary recommendations and health monitoring protocols, that may reproduce inequities or reduce engagement. These gaps limit identification of the structural racism‐related barriers most relevant to ADRD intervention effectiveness.

#### 2.2.2 Recommendations

Addressing structural racism in ADRD lifestyle intervention implementation requires a shift beyond inclusive recruitment alone toward redesigning intervention content, delivery, measurement, and evaluation to account for the persistent ways that structural forces uniquely disadvantage ethnoracially marginalized populations. Future studies should assess structural determinants that may influence intervention participation and effectiveness, including neighborhood segregation and health‐care access, as well as prior experiences of discrimination in research and clinical settings. Finally, greater attention is needed to the design of interventions to reduce assumptions based on model minority myths that may prevent addressing structural barriers unique to certain ethnoracial groups.

### 2.3 Structural genderism and related determinants

Structural genderism is the societal privileging of cisgender, heterosexual, and male identities through institutional policies, cultural norms, and systemic power dynamics.^38^ Operating alongside sexism, transphobia, and homophobia, structural genderism creates compounded barriers for women, lesbian, gay, bisexual, gay, transgender, queer, and other sexual minoritized (LGBTQ+) individuals, and gender‐diverse populations to achieve optimal health. Sexism, as a foundational element of structural genderism, contributes to significant disparities in caregiving burden, economic security, and health system engagement, all of which affect intervention participation and adherence.^39^, ^40^, ^41^ Women, particularly ethnoracially marginalized women and those in lower socioeconomic brackets, comprise the majority of informal caregivers, often with minimal support or compensation.^42^, ^43^ In a US sample, women accounted for 58% of informal caregivers,^44^ and among caregivers of community‐living persons with dementia, 32% reported high strain, only 11% had received training, and 18% had used respite care, with Black caregivers 69% less likely than White caregivers to use respite care.^45^ This under‐resourced caregiving labor, along with other stress imposed by interpersonal experiences of sexism and gendered discrimination that are largely invisible in health intervention planning, constrains the time, flexibility, and emotional capacity required to engage in structured health intervention programs.^41^ Yet, most structured interventions assume a degree of personal autonomy, time flexibility, and low caregiving burden.^46^

Structural genderism also operates through the erasure or marginalization of LGBTQ+ populations in aging and dementia research. Transgender and non‐binary older adults frequently experience health‐care discrimination, misgendering, and systemic exclusion from clinical settings, undermining trust and deterring participation in prevention‐focused trial.^47^, ^48^ Homophobia and heteronormative assumptions in intervention content (e.g., nuclear families’ references or gendered language in group sessions) may create unwelcoming or unsafe environments for LGBTQ+ participants.^49^ Moreover, LGBTQ+ older adults are more likely to live alone, less likely to have children, and more likely to rely on chosen families or community networks for support, which traditional models of intervention overlook.^50^, ^51^

Digital interventions such as home‐based cognitive training may also inadvertently marginalize gender‐diverse individuals through data privacy risks or the absence of inclusive design. Binary gender categories in online systems or intake forms can lead to misgendering, discomfort, or withdrawal from programs altogether. These biases are further compounded by policy‐level failures, including limited LGBTQ+ competency requirements in clinical trial protocols, or inadequate protections against discrimination in long‐term care and insurance coverage.^50^, ^52^

#### 2.3.1 Key knowledge gaps

Although sex and gender differences in ADRD burden and unequal caregiving responsibilities are increasingly recognized, few intervention studies examine how sexism, anti‐LGBTQ+ discrimination, caregiving expectations, or gendered health‐care discrimination affect intervention participation, adherence, effectiveness, or sustainability.

#### 2.3.2 Recommendations

ADRD intervention trials should evaluate whether flexible scheduling, respite support, remote participation options, childcare/eldercare support, or community partnership improve adherence among individuals with caregiving responsibilities, while intentionally recruitment of populations that are typically underrepresented in research on caregiving. Trials should also incorporate inclusive design features such as allowing participants to designate non‐family informants. Recruitment materials, intake forms, and intervention examples should use inclusive language and avoid binary or heteronormative assumptions, signaling to gender‐diverse individuals they are welcome in research agendas, service models, and health‐promoting interventions. Facilitators and study staff should receive training in LGBTQ+ competency and gender‐affirming communication. Study designs should be mindful of gendered caregiving burdens and provide supports that improve effectiveness of interventions.

### 2.4 Structural ableism

Structural ableism embeds assumed abilities and neurotypical norms into policies, research designs, and built environments, privileging certain physical, sensory, and cognitive capacities, implicitly defining health, participation, and success.^53^, ^54^ Many intervention protocols exclude individuals with visual, hearing, mobility, or cognitive impairments, narrowing generalizability and perpetuating inequities in access to evidence‐based prevention. Individuals with intellectual and developmental disabilities, who face significantly elevated risk for early‐onset dementia, are particularly vulnerable to this pattern of exclusion.^55^ Stigma associated with both disability and dementia contributes to lower treatment engagement and inclusion in community‐based programs.^56^

Exercise facilities often remain physically inaccessible to individuals with mobility impairments, chronic pain, or cardiorespiratory limitations. Even when accessible, staff may lack training in inclusive fitness training, adaptive equipment may be unavailable, and programming may not account for pacing modifications or non–weight‐bearing alternatives.^57^ Similarly, dietary guidance often presumes that participants can chop, cook, and shop independently, overlooking individuals with fine motor impairments, visual disabilities, or fatigue‐related conditions.^58^, ^59^

Transportation barriers complicate commitment to exercise, intervention appointments, and nutrition changes for individuals with disabilities living in transit‐inaccessible neighborhoods, especially in rural or under‐resourced areas.^60^, ^61^ Digital cognitive platforms may lack design features for sensory impairments, learning disabilities, or neurodivergence. Similarly, peer‐group formats or health coaching interactions may pose challenges for individuals with social communication disabilities, auditory processing difficulties, or psychiatric disabilities, particularly when facilitators are untrained in inclusive group dynamics.^53^ These environments may feel cognitively overwhelming or socially unsafe, contributing to reduced participation or program attrition.^62^

#### 2.4.1 Key knowledge gaps

Limited attention has been given to how accessible exercise facilities, adaptive physical activity prescriptions, accessible digital platforms, transportation support, and sensory accommodations, among others, affect intervention engagement and sustainability.

#### 2.4.2 Recommendations

The Alzheimer's Association has called for greater attention to stigma as a determinant of care avoidance and delayed intervention, particularly in populations with overlapping structurally driven vulnerabilities.^63^ Equitable ADRD intervention implementation requires addressing structural ableism as a foundational barrier. This includes expanding eligibility criteria, incorporating universal design in digital and in‐person formats, measuring accessibility and participation among individuals who are differently abled, and embedding anti‐ableism frameworks into research, policy, and funding structures.

### 2.5 Ethnocentrism, xenophobia, and related determinants

Ethnocentrism, which creates language and other cultural barriers, and xenophobia—defined as an intense dislike or fear of people from other countries, cultures, or of anything considered foreign—shape how individuals perceive, access, and engage with health interventions. In the United States, language discordance between health‐care providers and participants—often reinforced by policies and practices centered on English‐speaking, non‐Hispanic, Christian ethnic groups—can impair communication, reduce satisfaction, and lower intervention adherence among limited English proficiency populations.^64^ Religious and spiritual frameworks guide daily health behaviors, such as fasting practices, dietary restrictions, or sex‐specific physical activity constraints, which can conflict with prescriptive lifestyle changes.^65^ Additionally, xenophobia and perceived discrimination contribute to chronic stress and diminished trust in health‐care systems.^66^, ^67^ Frequently, dementia prevention trials exclude participants who are not English proficient.^68^ Moreover, anti‐immigrant sentiment can often result in the “chilling effect” in which individuals avoid services or public settings due to fear, well described in health services and policy research.^69^, ^70^ Comprehensive cultural adaptation and religious considerations are seldom systematically addressed unless the intervention is community initiated.^71^

#### 2.5.1 Knowledge gaps

Current studies provide limited guidance on how to adapt ADRD lifestyle interventions for immigrants, refugees, and those with limited English proficiency and religious diversity. Further, few interventions have directly measured or mitigated the effects of ethnocentrism, xenophobia, or anti‐immigrant sentiment on trial implementation effectiveness.

#### 2.5.2 Recommendations

Empirical studies have demonstrated that culturally adapted interventions can improve outcomes in minoritized groups,^72^, ^73^ supporting the feasibility and value of incorporating cultural variation into intervention design, as outlined in Table 1 and described further in the Discussion section. Multilingual recruitment materials, intervention content, cognitive assessments, and study communications are important to improve effectiveness in non‐native English speakers. Adaptable intervention content, including dietary recommendations that account for traditional foods rather than treating Western dietary patterns as the default standard for brain health, fasting practices, and religious restrictions, as well as physical activity options that respect gender‐specific norms, should be considered. Partnerships with immigrant‐serving organizations and faith communities, as examples, may improve trust and therefore implementation effectiveness. Future studies can better evaluate whether culturally adapted and linguistically concordant delivery improves intervention uptake, adherence, and effectiveness.

## 3 MICROSYSTEM‐LEVEL DETERMINANTS OF ADRD INTERVENTION IMPLEMENTATION EFFECTIVENESS

Acting as proximal manifestations of macrosystem forces, microsystem‐level determinants—or the immediate environments where individuals live, work, and interact—critically shape the feasibility, accessibility, and effectiveness of ADRD prevention interventions. Factors such as neighborhood and community environment directly affect community trust, psychological stress, and participation in health programs, acting as avenues through which macro‐level structures are actualized. The built environment, including green spaces, transportation access, and recreational opportunities, affect engagement in physical and social activities central to many ADRD lifestyle interventions. Workplace and occupational environments can either facilitate or hinder health‐promoting behaviors depending on stress levels, job security, and flexibility. Similarly, schools, as early‐life microsystems, shape cognitive, social, and health trajectories through literacy and education opportunities. Together, these microsystemic factors mediate how structural inequities manifest in day‐to‐day life, ultimately shaping who can engage with and benefit from ADRD lifestyle interventions. We expand on each of these microsystem‐level determinants in this section, highlighting their relevance to intervention design, accessibility, and sustainability across diverse populations (summarized in Table 1). Additional findings on poverty and income, physical activity, and education as microsystem‐level determinants are included in supporting information.

### 3.1 Neighborhood

#### 3.1.1 Nutrition, food access, and food environment

Healthy dietary patterns characterized by high intake of vegetables, fruits, nuts, fish, and unsaturated fats are consistently associated with slower cognitive decline, reduced risk of mild cognitive impairment, and lower ADRD mortality,^74^, ^75^ as they improve vascular health and metabolic regulation.^74^, ^76^, ^77^, ^78^ Yet, the capacity of individuals to adopt and sustain these diets is shaped by constraints within their immediate environment.

Food insecurity, defined as restricted access to adequate nutritious food due to constrained economic resources (US Department of Agriculture Economic Research Service),^79^ is a major barrier to implementing lifestyle modification interventions.^80^ Food insecurity impacts > 10% of US households, and disproportionately burdens Black, Hispanic, and Native communities, as well as low‐income rural older adults.^5^, ^6^, ^11^ Affected individuals often face limited access to full‐service grocery stores, greater exposure to convenience stores and fast food (“food swamps”), longer travel distances (“food deserts” or areas where high‐quality food is difficult to access), and higher food prices, reducing sustained adherence to dietary changes.^81^ Food insecurity is associated with lower vigorous physical activity due to lower energy availability, fewer recreational resources, and competing financial or caregiving demands.^82^, ^83^, ^84^, ^85^ Food deserts can increase the risk of poor diet and obesity, upstream factors to ADRD.^85^, ^86^ Food insecurity also directly affects cognition^87^ and exacerbates chronic medical conditions that reduce capacity to engage in cognitive training^88^ and health monitoring.^89^ This cumulative disadvantage suggests that interventions focused only on diet adherence without addressing food availability and affordability may have limited effectiveness and may exacerbate inequities. Implementation and adherence to ADRD lifestyle interventions may be lower among those with food insecurity, as better diet quality is associated with greater participation in these activities.^90^, ^91^

Food access programs may strengthen or complement diet interventions in ADRD prevention. Meal preparation and delivery assistance programs provide nutrition and cognitive health education, support prevention of unintentional weight loss, and may be particularly relevant for individuals with early cognitive symptoms or rural residence.^92^ Federal programs such as the Supplemental Nutrition Assistance Program (SNAP) reduce food insecurity and improve nutrition intake among low‐income households.^93^, ^94^ Community‐tailored approaches, including culturally adapted mobile markets and partnerships with food banks, further improve the feasibility and sustainability of dementia‐prevention strategies.^20^

*Knowledge gaps*: While there is substantial evidence linking diet quality to ADRD across culturally diverse populations, few studies have empirically examined how food access—food insecurity, food deserts/swamps, food affordability, and culturally specific dietary practices—may operate to influence uptake and effectiveness of ADRD lifestyle interventions. There is likewise a dearth of literature examining how food access may variably interact with other microsystem factors including health‐care use and caregiving burden to influence engagement in ADRD lifestyle interventions across diverse populations.^92^, ^95^

*Recommendations*: Interventions to address healthy eating should (1) enhance equitable access to nutritious foods via collaborations with programs like SNAP or Meals on Wheels and community food systems, (2) guarantee cultural adaptation of dietary recommendations, and (3) incorporate dietary guidance with lifestyle interventions in community contexts. These adaptations may reduce stress pathways associated with food insecurity and help embed interventions within trusted community networks, which is crucial for sustainability. Future studies should test whether such approaches increase the sustainability of ADRD lifestyle interventions across culturally diverse populations.

#### 3.1.2 Pollution

Environmental pollution includes human‐generated contaminants into the air, water, soil, and built environment, as well as increased noise and artificial light exposure from industrial activity and high urbanization. Strong evidence links environmental pollutants to poor health, including ADRD and related risk factors,^96^, ^97^, ^98^ while emerging evidence suggests noise and light pollution may be associated with ADRD through mechanisms such as sleep disruption, chronic stress, and cardiometabolic disease.^99^, ^100^, ^101^, ^102^, ^103^ In the United States, uneven enforcement of zoning, environmental, and health policies places pollution sources closer to communities that are socioeconomically disadvantaged and predominantly Native, Black, Hispanic, and Asian.^97^, ^104^, ^105^, ^106^, ^107^

Although natural experiments suggest that reducing pollution may lower risk for ADRD,^108^, ^109^ clinical trials rarely account for pollution as a barrier to participation. Observational studies suggest pollution may indirectly reduce the adherence to and effectiveness of lifestyle modifications. For instance, greater air pollution discourages physical activity and outdoor leisure time, particularly among women,^110^ and results are mixed as to whether aerobic intake in high air pollution is related to higher rates of cardiometabolic disease.^110^, ^111^, ^112^, ^113^

Less is known about pollution's effects on dietary adherence. However, food, soil, and water contamination (e.g., pesticides, heavy metals, endocrine‐disrupting chemicals, plastic packaging that degrades upon heating) may blunt the impact of interventions.^106^, ^114^ These risks are amplified in highly industrialized areas where access to fresh foods is limited, and environmental health literacy is lower. ^106^, ^114^, ^115^ Studies have not assessed noise and light pollution effects on lifestyle interventions; however, these are likely to have indirect effects through sleep disruption. Low sleep quality is associated with impaired athletic performance,^116^, ^117^, ^118^ increased caloric intake,^119^, ^120^ impaired glycemic control,^121^ and reduced executive function and attention,^122^, ^123^, ^124^, ^125^ each of which would present barriers to engaging in physical exercise, nutrition change, and cognitive exercise.

*Knowledge gaps*: Pollution is rarely incorporated into ADRD lifestyle intervention research despite its relevance to several ADRD risk factors including physical activity, sleep, cardiometabolic health, diet quality, stress, and cognition.

*Recommendations*: Future studies should explicitly study pollution's role in moderating participants’ adherence to and benefit from ADRD‐related lifestyle interventions, especially among populations in high pollution settings. Further, studies should consider whether water/soil contamination, pesticide exposure, noise pollution, or artificial light exposure moderate intervention effectiveness. Trials should test mitigation strategies, including air quality monitoring, indoor exercise options, education on foodborne contaminants, and tools to reduce nighttime light and noise exposure.^114^

#### 3.1.3 Greenspace and built environment

Proximity to greenspace—parks, tree canopy coverage, natural outdoor areas, and community green corridors—has physiological benefits^126^, ^127^, ^128^, ^129^ and has been associated specifically with reduced risk for vascular cognitive impairment,^129^ underscoring its relevance as an environmental condition that supports cardiometabolic regulation, a central target of ADRD lifestyle interventions. Beyond physical health, greenspace exposure influences cognitive and emotional processes that may moderate intervention response. Natural environments reduce physiological stress markers, improve mood, enhance attentional capacity, and lower allostatic load.^130^, ^131^ These mechanisms overlap with pathways targeted in ADRD lifestyle interventions. In this way, greenspace functions as a supportive microsystem context that may amplify the effectiveness of intervention activities by reducing competing psychological and physiological burdens.

Lower income and ethnoracially minoritized neighborhoods, particularly those historically subjected to redlining and other discriminatory zoning and housing practices, consistently have significantly less greenspace, fewer street trees, and lower quality natural spaces.^132^, ^133^, ^134^ These inequities reflect macrosystem‐level forces, such as historical disinvestment, discriminatory lending, and segregation, that manifest at the microsystem level as environmental deprivation. Even when individuals are highly motivated and interventions are well designed, environmental constraints may limit the feasibility or potential impact of lifestyle‐change strategies, and assumptions of universal access to greenspace may inadvertently reduce equitable participation and adherence. Interventions that encourage walking, stress reduction practices, or outdoor engagement may be more feasible and beneficial for individuals in greener environments.

*Knowledge gaps*: Existing research rarely evaluates whether neighborhood greenspace or built environment features influence implementation effectiveness of lifestyle interventions, particularly in relation to physical activity and social engagement.

*Recommendations*: Integrating built environment considerations into ADRD intervention planning, such as offering indoor or virtual alternatives (e.g., indoor walking routes, home‐based exercises), acknowledging environmental disparities in adherence expectations, and partnering with community organizations to identify safe natural areas, are essential for achieving equitable implementation and maximizing cognitive health benefits across diverse populations.

#### 3.1.4 Transportation

Regular attendance to structured interventions depends on access to safe, affordable, and frequent transit, conditions not met in many low‐income, rural, or ethnoracially segregated neighborhoods.^6^, ^80^, ^135^ Therefore, transportation barriers can substantially reduce participation and retention across dementia prevention interventions for these groups.^136^, ^137^, ^138^, ^139^, ^140^ Physical activity and health monitoring interventions are especially affected when participants must travel to centralized locations, while dietary and cognitive programs can more readily be adapted for remote delivery. As described above, in the absence of dietary programs, the ability to improve dietary habits hinges on access to affordable, healthy foods, which often can be located in distant or affluent neighborhoods, compounding structural barriers for those without reliable transit.^141^ Online formats help eliminate travel needs but introduce new inequities related to digital access and literacy, which disproportionately affect older adults from disadvantaged backgrounds.^142^, ^143^ These findings are reinforced by multiple studies conducted in underrepresented populations, which consistently highlight the value of such logistical adaptations in facilitating engagement.^49^, ^136^, ^144^, ^145^, ^146^, ^147^

*Knowledge gaps*: Despite promising adaptations for specific lifestyle interventions that allow for online formats and remote delivery, transportation‐related implementation outcomes are still rarely reported in a standardized way, echoing broader critiques of methodological inconsistency in recruitment and retention reporting across ADRD research.^42^

*Recommendations*: To ensure equitable access, future programs should embed transportation support (e.g., vouchers, shuttle services) and delivery flexibility (such as hybrid or home‐based visits and online formats) into the design of ADRD lifestyle interventions. For participants with mobility limitations or rural residence, home‐based or community‐based delivery should be considered from the outset rather than treated as a secondary accommodation. It may also be worthwhile to understand if and how delivery flexibility may introduce digital access and literacy barriers, and remote delivery should be paired with digital access support to avoid substituting transportation inequity with digital inequity. Finally, studies should monitor and report transportation‐related barriers, missed visits due to travel difficulties, travel time, mode of transportation, and costs to participants to better understand barriers to implementation effectiveness.

#### 3.1.5 Housing

Housing precariousness, sometimes referred to as housing adequacy,^148^ has been operationalized as a multi‐domain construct including affordability, security/stability, quality and facilities, tenure type, and access to services.^149^, ^150^ Housing precariousness can affect a person's likelihood of being recruited into clinical trials and engagement in targeted population‐level interventions, as housing stability can constitute an exclusion criterion.^12^ Recruitment efforts that rely on responses through letters and postcards, for example, exclude individuals who experience homelessness.

Housing precariousness may also reduce the effectiveness of ADRD interventions by impairing participants’ ability to adhere to intervention protocols. Barriers to adherence can arise from housing affordability, quality, and residential stability, all of which have been associated with poorer engagement in health behaviors and chronic disease self‐management.^151^, ^152^ Individuals experiencing high housing cost burdens may have fewer financial resources available for other essential needs, limiting their ability to adhere to diet‐based interventions, maintain gym memberships, or participate in other interventions that entail financial costs.^151^ Consistent with this, budgetary constraints and the high cost of healthy foods have been identified as key barriers to adherence to dietary patterns such as the MIND diet.^153^, ^154^ In addition, living in crowded or noisy housing environments may impede engagement with cognitive training interventions, as residential crowding and environmental noise have been linked to poorer cognitive performance and reduced attentional capacity.^155^, ^156^ Finally, poor housing quality, including limited or inadequate kitchen facilities, may restrict individuals’ ability to prepare healthy meals, further undermining adherence to diet‐focused intervention components.^157^

*Knowledge gaps*: Overall, there has been a dearth of studies examining the intersection of housing precariousness and lifestyle intervention effectiveness. It is still not well understood how housing affordability, residential instability, homelessness, inadequate kitchen facilities, or limited access to services affect not only recruitment but intervention benefits. This understudied area may be particularly important for identifying opportunities to advance intervention equity, as sexual, gender, and ethnoracially minoritized populations are more likely to experience housing precariousness.

*Recommendations*: Recruitment strategies should not rely exclusively on mailing addresses or traditional health care–based contact methods. Intervention protocols should consider flexible participation options for individuals with unstable housing, including community‐based delivery, mobile outreach, meal support, and alternative communication methods. Providing alternatives for those who do not have adequate kitchen facilities, cooking equipment, and food storage capacities to enable diet‐based intervention effectiveness is also warranted. Future research would advance the evidence base for such design features by directly examining how housing precariousness influences participation in ADRD lifestyle interventions.

### 3.2 Community and social connections

#### 3.2.1 Policing and the carceral system

Although direct evidence linking policing and incarceration to ADRD is limited, research suggests that incarceration and aggressive or discriminatory policing practices contribute to chronic stress, trauma, and institutional mistrust,^158^, ^159^, ^160^, ^161^ which in turn are associated with cognitive decline and higher prevalence of ADRD in aging prison populations.^162^, ^163^ These experiences disproportionately affect ethnoracially minoritized communities, reflecting broader structural inequities in the criminal legal system.^164^

Yet, few ADRD interventions explicitly account for how interactions with carceral systems may undermine implementation. Individuals on probation or parole may face restrictions that limit their ability to attend regular group sessions, access certain community facilities, or travel to intervention sites. Adults reentering society after incarceration often encounter unstable housing, limited internet access, food insecurity, fragmented health‐care continuity, and competing survival priorities such as securing identification documents or employment.^164^, ^165^, ^166^ These environmental constraints can make engagement with multicomponent interventions nearly impossible without structural support.

*Knowledge gaps*: The effects of policing, incarceration, probation, parole, and re‐entry on ADRD lifestyle intervention implementation remain largely unstudied. While there are clear associations of barriers to participation after incarceration with discriminatory policing, including stress, trauma, mistrust, and health‐care disruption, among others, few ADRD intervention studies account for these exposures as barriers. Individuals affected by the carceral system may face many of the barriers previously described, including unstable housing, food insecurity, limited internet access, and restricted movement that make attendance difficult, thus affecting implementation effectiveness; however, these relationships have not been tested.

*Recommendations*: To promote equitable and sustainable participation, ADRD interventions must integrate strategies tailored to reentry populations. Potential approaches include offering flexible or decentralized delivery (e.g., mobile health units, virtual programming), partnering with reentry and community‐based organizations, embedding trauma‐informed practices, coordinating with case managers or probation officers to navigate logistical barriers, and providing safe, accessible spaces for participation. Embedding cognitive health promotion within broader reentry support ecosystems, rather than requiring individuals to access siloed services independently, may also reduce the microsystem realities that disproportionately constrain ethnoracially minoritized populations.

#### 3.2.2 Household and family

Family and household contexts exert a substantial influence on the development and maintenance of health behaviors, including physical activity, dietary practices, sleep, and other lifestyle‐related habits.^167^, ^168^ Accordingly, they can play a critical role in the initiation, uptake, and sustainment of behavior change interventions.^167^ Among middle‐aged adults, who often face competing occupational, caregiving, and social demands that may impede sustained behavior change, intervention designs that incorporate family or household members have been associated with improved adherence to programs targeting physical health, cognitive health, and nutrition. ^169^

Even still, the benefits of familial participation may vary across populations. Culturally patterned caregiving expectations, family role obligations, health beliefs, and stigma may shape a household member's willingness or capacity to participate in structured interventions intended to support a loved one's health behavior change.^167^, ^170^ Sexual and gender minoritized individuals may be especially affected by household dynamics in lifestyle intervention contexts because these populations experience elevated exposure to family disruption, housing instability, and economic insecurity across the life course, including midlife.^170^

*Knowledge gaps*: Few intervention trials systematically assess familial or household dynamics, limiting the field's ability to identify and mitigate family‐related barriers to participation or, conversely, to leverage household resources that may help sustain behavior change.^169^ The literature is also sparse regarding how family or household context influences research participation in health behavior interventions, particularly for ADRD studies.^167^ This gap is especially pronounced for racially, ethnically, and linguistically minoritized populations; although some studies have examined exercise or lifestyle interventions in rural or underserved communities, these studies rarely center the perspectives of minoritized groups.^169^, ^171^ This omission limits the generalizability of existing findings and underscores the need for more inclusive, culturally responsive research on how household and family factors shape the implementation of ADRD interventions.

*Recommendations*: Research should measure household composition, family health beliefs, cultural norms, and competing obligations to determine how and whether household and family inclusion in interventions promotes effectiveness in lifestyle interventions. Further, studies may consider oversampling diverse household structures to evaluate whether family‐/peer‐supported, and individually delivered models differ in implementation effectiveness outcome. Intervention models should offer flexible support structures, allowing participants to designate family members, friends, chosen family, peers, or community health workers as support partners. Trials such as POINTER have demonstrated the utility of this approach. Future research opportunities include examining how cultural, socioeconomic, and geographic factors influence family/household member involvement in the implementation and effectiveness of ADRD‐focused interventions.

#### 3.2.3 Social networks and isolation

Social support is both a motivational catalyst and an adherence mechanism, particularly in interventions requiring sustained behavioral change.^172^, ^173^, ^174^ The social component of health behavioral change programs, like cognitive stimulation, contributes to better adherence and motivation, offering problem solving and discussion opportunities through conversation and shared routines.^175^, ^176^ Engagement often declines over time when attempting to conduct this type of activity alone, particularly if the technology is unfamiliar or tasks become repetitively self‐directed.^172^, ^173^

Nutrition interventions are also more effective when they incorporate social support, in part through encouragement and emotional reinforcement when progress is slow or setbacks occur.^173^, ^174^, ^177^ Peer‐delivered or “buddy” interventions have further demonstrated that structured social support can enhance dietary adherence and physical activity.^178^ Moreover, sharing health goals in a group context fosters communal accountability and can reduce the stigma associated with chronic conditions.^179^, ^180^ Without these relational supports, health monitoring can feel burdensome or even pointless, limiting long‐term adherence.

*Knowledge gaps*: While there is evidence to support the benefits of social support on ADRD lifestyle intervention effectiveness and outcomes, the field lacks sufficient work on how to tailor social‐support components to diverse community contexts and to those who do not have robust support systems.

*Recommendations*: ADRD lifestyle interventions should consider programs that embed opportunities for peer connection to cultivate a sense of belonging that buffers against stress, isolation, and withdrawal to improve implementation effectiveness.^181^ As an example, incorporating digital literacy resources to reduce technology‐driven isolation and inequities in remote program delivery may improve implementation sustainability. For those who cannot or do not want to participate in group settings, interventions should offer alternative forms of support, such as peer navigators, community health workers, telephone‐based methods, or digital social platforms with accessibility safeguards. Further empirical research on ADRD lifestyle interventions should incorporate measures of social isolation, loneliness, network size/quality, and perceived belonging to better understand the specific pathways through which these factors may influence implementation effectiveness.

#### 3.2.4 Gentrification

Gentrification is defined as the “process of neighborhood [alteration] that includes economic change in a historically disinvested neighborhood—by means of real estate investment and new higher‐income residents moving in—as well as demographic change” that influences income or education levels and/or the ethnoracial makeup of residents within a community.^182^ Historically marginalized populations frequently experience the greatest consequences, such as displacement, loss of social cohesion and connection, food insecurity, poorer physical and mental well‐being, less access to age‐appropriate resources and recreation, and more chronic health difficulties.^183^, ^184^, ^185^, ^186^, ^187^, ^188^ Though significant gaps still exist in our understanding of the association between gentrification and ADRD, especially regarding the pathways and underlying mechanisms, there is emerging evidence that gentrification is associated with cognition or cognitive‐related factors in older adults, especially those who identify from ethnoracially diverse groups.^189^, ^190^ These early findings are not only crucial for establishing foundational knowledge of how gentrification relates to non‐pathological and pathological aging (i.e., ADRD), but also highlights the potential barriers to engaging in interventions to maintain cognitive health.

The effectiveness of lifestyle interventions may be attenuated by the complex and intersecting effects of gentrification. Gentrification can limit the ability to fully engage in lifestyle changes through multiple pathways.^11^, ^12^ One prominent mechanism is residential displacement, as rising housing costs may force individuals out of neighborhoods in which they have lived for decades, disrupting established social networks and altering access to familiar, age‐appropriate resources, including community centers and affordable food outlets.^183^, ^184^, ^191^ In addition, exposure to gentrification‐related stressors can lead to higher levels of chronic psychological stress and physical and mental health conditions, which may contribute to accelerated aging or premature mortality and, at minimum, impede sustained engagement in interventions—an issue that may be particularly salient for racially and ethnically diverse populations.^192^, ^193^, ^194^, ^195^, ^196^ Finally, the erosion of neighborhood infrastructure that can accompany gentrification may create structural barriers to the implementation and delivery of health behavior interventions, although programs exist that have successfully implemented cognitive health interventions in gentrified contexts by leveraging asset‐based frameworks.^197^

*Knowledge gaps*: Despite the potential pathways described above, no discernable studies have empirically examined the role of gentrification in shaping ADRD lifestyle intervention implementation, nor how any impact of gentrification on intervention effectiveness may operate among the ethnoracially minoritized groups most likely to be displaced.

*Recommendations*: ADRD lifestyle interventions should partner with trusted community organizations, tenant groups, senior centers, and neighborhood coalitions to preserve access in rapidly changing neighborhoods. Asset‐based approaches that leverage existing community strengths may help sustain intervention engagement.

### 3.3 Workplace/occupation

Workplace characteristics, including job complexity and schedule flexibility, can shape opportunities to maintain cognitive health and influence the feasibility of implementing ADRD interventions. High‐demand or inflexible occupations may impose structural and psychosocial constraints that limit individuals’ ability to engage in health‐promoting behaviors, such as maintaining balanced nutrition, participating in regular physical activity, or attending medical appointments.^198^, ^199^ These constraints are particularly pronounced for caregivers, whose occupational and caregiving burdens often overlap, leading to reduced engagement in preventive health behaviors,^200^ missed medical appointments,^201^ and compromised nutrition and physical activity.^202^ Collectively, these findings suggest that occupational limitations on personal bandwidth—driven by time scarcity, fatigue, and emotional depletion—represent a critical rate‐limiting factor in the adoption and sustainability of health interventions.

*Knowledge gaps*: A growing body of evidence demonstrates that workplace‐based interventions are among the most effective approaches for promoting exercise and nutrition behaviors among adults in the United States.^203^, ^204^ Despite this evidence, substantial gaps remain, including limited understanding of the extent to which social and cognitive interventions can be successfully implemented across diverse occupational contexts—particularly in settings characterized by high job demands or limited flexibility. This uncertainty is compounded by evidence that complex or cognitively demanding occupations may mitigate ADRD risk, potentially obscuring the need for or benefits of additional intervention.^205^, ^206^ Moreover, studies examining the intersection of workplace exposures, nutrition, and physical activity remain sparse, especially in occupational settings where health‐promoting behaviors are not institutionally incentivized, highlighting an important opportunity for future research.

*Recommendations*: Especially as it pertains to ADRD intervention trials targeting prevention or prodromal/preclinical phases of ADRD, where participants may still be working, intervention designs should include flexible schedules, remote options, or brief intervention modules. Furthermore, to the extent possible, working with participants to engage in workplace‐based interventions may promote sustainability in ADRD lifestyle interventions. These approaches may mitigate some of the occupation‐related barriers to sustaining lifestyle changes experienced by marginalized populations and enable more equitable outcomes of ADRD lifestyle interventions.

### 3.4 Literacy

The term “literacy” covers different aspects of functioning, including fundamental literacy (reading and writing skills), digital literacy (ability to effectively, safely, and responsibly use digital technologies to find, evaluate, create, and communicate information), and health literacy (the ability to access, comprehend, appraise, and apply health information). Although most common in those with a high school education or less, low literacy also occurs among those with more than a high school education,^207^ and is marked by increasing racial/ethnic disparities.^208^ Additional details on the impact of education in ADRD intervention effectiveness can be found in Supplemental materials. In ADRD prevention research, literacy is rarely measured or reported, and education is usually categorized by high school, college/no college, and graduate degree, which may not reflect true levels of literacy or quality of education.^11^ Yet, health literacy has been linked to adherence in lifestyle interventions including healthy diet habits,^209^, ^210^ while numeracy, or the ability to understand/work with numbers, has been associated with poor understanding of nutrition labels.^211^, ^212^

Intervention implementation barriers may occur due to methods of inviting participants (e.g., using letters and sending questionnaires) or requirements for written informed consent. Although most institutional review boards require written consent forms to be at a sixth to eighth grade reading level, there is limited evidence on effective informed consent for low‐literacy populations.^213^ Other sources of bias include cognitive and screening tests that can result in greater frequency of misdiagnosis among less educated or low‐literacy participants, questionnaires or food logs that may be difficult to fill out, or informational materials in written form that may be more difficult to understand. Likewise, web‐based cognitive training platforms pose challenges for those with limited digital or fundamental literacy.^212^

*Knowledge gaps*: Evidence on how literacy affects various ADRD lifestyle intervention modalities is lacking. Collecting participants’ literacy levels would help determine whether face‐to‐face assistance from health coaches can help overcome barriers that populations with lower literacy face in interpreting and using ADRD intervention information, as is shown in diabetes research, in which peer health coaches can promote diabetes control in culturally, educationally, and linguistically diverse patient groups.^214^

*Recommendations*: While education attainment is often used as proxy for literacy, it does not reliably capture reading abilities, health literacy, numeracy, and digital literacy, among others. ADRD intervention trials should formally assess the literacy components mentioned above and evaluate whether these factors modify intervention uptake and sustainability. Intervention materials should be written in plain language and supplemented with oral explanations, visual aids, teach‐back methods, multilingual materials, and culturally relevant examples. Digital platforms should be designed for users with varying literacy and technology experience. Health coaches, peer navigators, and community health workers may help participants interpret and apply intervention information, particularly in educationally and linguistically diverse communities. Additional studies are needed to determine whether other delivery modalities, like visual tools or peer navigation, can reduce literacy‐related barriers. Finally, food logs, questionnaires, and cognitive training tasks should be evaluated for literacy‐related burden and potential measurement bias.

### 3.5 Health‐care access

Disparities in health‐care access and quality disproportionately affect individuals with lower incomes, those living in rural areas, linguistically diverse or non‐English speaking populations, and people from diverse cultural, racial, or historically minoritized backgrounds.^215^, ^216^, ^217^, ^218^, ^219^ Individuals facing these barriers or receiving lower quality care are more likely to have comorbid conditions and poorer overall health, a pattern particularly pronounced among ethnoracially minoritized groups,^220^, ^221^, ^222^ which may limit their ability to engage in lifestyle interventions on ADRD. Health monitoring, a key component of effective ADRD prevention, requires reliable access to health‐care professionals and facilities, which in turn rely on a functional system–population interface.

Insurance coverage type also shapes health‐care access. While Medicare covers many ADRD‐related services and treatments for individuals ≥ 65 years of age, out‐of‐pocket health‐care costs remain a significant impediment to health‐care use, which may deter or interfere with intervention participation.^223^ Lifestyle interventions hold promise for facilitating health‐care access and improving the reach of ADRD interventions in underserved communities. However, building an infrastructure to equitably implement sustainable health practices that promote cognitive health across diverse populations after the conclusion of an intervention trial requires supportive health‐care policies and broader federal and state commitment—factors that are, as earlier described, substantially influenced by macrosystem‐level dynamics.

*Knowledge gap*: Healthcare‐related factors that support the long‐term maintenance of ADRD lifestyle interventions remain understudied.

*Recommendations*: Lifestyle interventions for ADRD should incorporate health‐system navigation, linkage to primary care, and referral pathways for unmet health needs. Health monitoring components should also be designed in consideration of individuals with limited access to routine care. Interventions should incorporate feasible and affordable models for ongoing monitoring and care, including in‐home care programs, mobile clinics, community health worker partnerships, and access to multidisciplinary providers. Trials should also consider solutions like subsidized devices, expanded coverage, tele‐audiology, and community‐based screening in underserved neighborhoods. Provider and community health education should also be integrated to strengthen ADRD awareness, improve referral pathways, and support sustained intervention engagement beyond the trial setting. Studies should examine how health care–related barriers, including insurance coverage, out‐of‐pocket costs, rurality, and others, influence sustained adherence. Additional work is also needed to determine how ADRD lifestyle interventions can be integrated into routine care, Federally Qualified Health Centers, community health systems, and payer‐supported preventive care models.

## 4 DISCUSSION

This narrative synthesis explores how multilevel structural and social determinants, from macro‐level forces like structural racism and genderism to microsystem factors such as poverty, policing, and community context, influence the implementation effectiveness of ADRD lifestyle interventions. Our review demonstrates that determinants at both macro and micro levels of society shape availability, uptake, and sustainability of behavioral interventions by creating differential access, engagement, and adherence pathways. We found that there is promising evidence for the effective implementation of lifestyle interventions targeting physical activity, nutrition, cognitive and social engagement, and health monitoring. Particularly, randomized trials have shown successful outcomes through intentional recruitment of individuals from minoritized populations. Nonetheless, systemic inequities across multilevel and interdependent social, economic, and institutional contexts continue to create substantial opportunities for increasing intervention effectiveness and equitable delivery.

In the following sections, we discuss theories that offer insight into the pathways we have described linking macrosystem and microsystem factors to the behavior change techniques implemented within many lifestyle interventions; we further detail examples of intervention studies that successfully address S/SDOH; we summarize key knowledge gaps related to the role of S/SDOH in ADRD intervention literature; and we summarize our recommendations on opportunities for study design, future research, advocacy, and collaboration to advance equity in population cognitive health.

### 4.1 Proposed mechanisms

Psychosocial stress may serve as a critical mechanism linking macro‐ and microsystem determinants to lifestyle intervention outcomes. Often measured as an individual process, stress can also be understood as a structural process, situated downstream of macrolevel factors but upstream of behaviors and physiological outcomes. While stressors can be acute or temporary, they are frequently chronic and systematized in marginalized populations due to inequitable social gradients in income, health‐care access, and housing. Cumulative stress theories highlight how chronic psychosocial stressors are compounded by limited resources to buffer their impact, disproportionately affecting socioeconomically marginalized groups, sexual and gender minorities, and ethnoracially minoritized populations.^224^, ^225^, ^226^ Substantial evidence connects psychosocial stress to ADRD risk factors like poor sleep, depression, and vascular dysfunction,^227^, ^228^ while undermining behavioral intervention uptake and maintenance. Observational and intervention studies show that high stress can cause reduced treatment adherence and greater attrition.^229^, ^230^ Thus, to equitably and sustainably implement ADRD lifestyle interventions, intervention design must specifically integrate strategies to address psychosocial stress, both by identifying and directly mitigating the stressors (i.e., transportation barriers, food insecurity, experiences of stigmatization and discrimination in health care) and by screening for psychosocial stress to connect participants with stress‐mitigating resources to which they may otherwise lack access.

Theories of health behavior change offer further insights into how macro‐ and microsystem factors influence long‐term intervention uptake and sustainability. Models such as Ecological Systems Theory on which this review is based emphasize that behavior is shaped not only by individual motivation and knowledge but also by social context, resource availability, and structural barriers.^9^ For instance, perceived susceptibility and self‐efficacy—core components in behavior change^231^—are constrained by factors like economic hardship, social isolation, or neighborhood disinvestment.^232^, ^233^ Furthermore, sustained behavior change relies heavily on social support networks and environments facilitating maintenance of the desired behavior,^234^ which are often less accessible for ethnoracially and socioeconomically marginalized groups as a result of macrosystem forces shaping the distribution of these resources. These frameworks suggest that interventions tailored to address systemic constraints and build on community strengths are more likely to succeed in diverse populations.

### 4.2 Examples of promising intervention strategies

Promising opportunities exist to enhance equity in ADRD lifestyle intervention implementation by leveraging resilience and resources within marginalized communities through culturally responsive recruitment strategies, community‐based participatory research (CBPR), and tailored intervention models grounded in evidence‐based theory. Effective recruitment in marginalized populations often involves partnering with trusted community organizations, such as faith‐based groups, senior centers, and local advocacy organizations, before research studies are created or funding is secured to build rapport and overcome mistrust rooted in historical injustices. For instance, the REACH (Resources for Enhancing Alzheimer's Caregiver Health) studies successfully engaged diverse caregivers, including Black and Hispanic populations, by employing bilingual staff and community liaisons embedded within local networks.^235^ Similarly, recruitment efforts using community health workers or peer navigators have shown promise in increasing participation among Latinx and Indigenous older adults by bridging cultural and linguistic gaps.^236^, ^237^

CBPR approaches, which involve community members as equal partners in research design, implementation, and dissemination, have demonstrated enhanced relevance, trust, and sustainability of interventions. For example, the Healthy Aging Brain Care (HABC) model was co‐developed with Black elders to address culturally specific barriers to cognitive health promotion, resulting in increased uptake of memory screening and lifestyle interventions.^238^ The Cognitive and Emotional Health Project incorporated CBPR principles to tailor cognitive training programs to Native American elders, incorporating traditional storytelling and culturally congruent activities, which improved engagement and perceived relevance.^239^

Beyond recruitment and design, culturally tailored interventions have improved behavioral outcomes by aligning content and delivery with participant lived experiences. The Memory Keepers program, targeting Black communities, integrates spirituality, communal values, and culturally preferred communication styles to promote cognitive health, resulting in better adherence to physical activity and nutrition goals compared to standard interventions.^240^ Similarly, nutrition interventions adapted for Hispanic elders incorporate traditional foods and cooking methods to enhance acceptability and adherence.^241^ Cognitive training delivered through community centers and facilitated by trusted local leaders has increased participation among LGBTQ+ older adults when facilitators receive training in gender‐affirming care and sensitivity to the unique stressors faced by sexual and gender minoritized persons. These examples highlight the necessity of moving beyond one‐size‐fits‐all models to community‐engaged, culturally tailored intervention frameworks that leverage existing social networks, build trust, and address structural barriers. By centering community strengths, ADRD lifestyle interventions can achieve greater relevance, engagement, and sustainability in historically marginalized populations.

Without intentional adaptation and structural support, even the most rigorously designed interventions risk reproducing the very disparities they aim to address. Fortunately, there are scaffoldings of ways to help generate more equitable clinical guidelines and policy. The Public Health Critical Race Praxis (PHCRP) framework provides a path forward by inviting researchers to interrogate and challenge how racism operates in the research process through understanding the sociohistorical context of racialization, deconstructing disciplinary biases, engaging affected communities in study design, and translating research into action that disrupts structural inequities.^242^ Studies like POINTER offer the opportunity to test these theory‐grounded approaches by using real‐world data to assess how macro‐ and microstructural factors impact trial design, who is enrolled, how participants engage with the study interventions, and how findings can be translated into clinical guidelines and policy in a way that does not reinforce inequity. With these efforts, evidence‐based interventions can reduce risk of perpetuating exclusion or misalignment with the lived experiences of all marginalized populations.

#### 4.2.1 Key knowledge gaps

Despite growing recognition of systemic biases and examples of successful efforts to address them, evidence gaps remain for strategies to facilitate understanding and targeting macrostructural barriers and their intersections in ADRD lifestyle interventions. For example, participation stratified not only by socioeconomic status and race or ethnicity, but also by gender identity and sexual orientation, necessitates inclusive designs that challenge normative assumptions often embedded in lifestyle intervention models. Yet, the impact of this intersectional group membership on intervention effectiveness is rarely examined. The impact of structural racism on cognitive training and health monitoring interventions also remains understudied, though frameworks like PHCRP offer methodological tools to advance this work.^242^ Moreover, macrosystem factors such as xenophobia and ethnocentrism are rarely examined in relation to intervention adherence, highlighting the need for research that specifically examines the impact of culturally responsive protocols and evaluation on intervention access and adherence. Community context also demands greater attention; neighborhood‐level data and participatory design remain underused despite evidence linking environment to health behaviors.

At a microstructural level, significant gaps exist in research on areas such as income/poverty, literacy, transportation, greenspace, housing, and workplace/occupation that have not been thoroughly examined when considering design and implementation of lifestyle interventions to prevent ADRD. Notably, when available, most studies to date have interrogated behavioral interventions like nutrition or diet and physical exercise, while far fewer studies have focused on cognitive or social engagement and health monitoring interventions. Other considerations, including community context across microstructures—such as the impact of policing and carceral systems on social connection, food access, or health‐care access—are likewise understudied.

Community‐engaged interventions, such as those implemented through senior centers or neighborhood‐based organizations, have consistently reported better participant engagement and sustained behavior change compared to centralized, academic medical centers.^243^ Furthermore, place‐based health interventions that incorporate community health workers or local leaders show increased trust and uptake, particularly in racially marginalized communities.^244^ Yet, many studies fail to include neighborhood/community‐related granular data on microsystem characteristics or measure their direct influence on implementation success. Tools like Geographic Information System–based resource mapping or neighborhood deprivation indices are infrequently used despite their availability. Data from existing studies (e.g., POINTER) offers an opportunity to assess neighborhood‐level variation in implementation and to pilot context‐responsive delivery models that promote equity in ADRD prevention and can be leveraged to address some of these research gaps. While a large and growing body of evidence links macro‐ and microlevel determinants to lifestyle intervention implementation and effectiveness, there remains several areas where additional research can further our understanding of these relationships in actionable ways.

#### 4.2.2 Specific recommendations

Based on our findings, we provide recommendations for clinicians, researchers, interventionists, public health practitioners, and policy makers to strengthen the relevance, sustainability, and effectiveness of lifestyle interventions across diverse populations and to promote equitable outcomes in ADRD prevention trials. Organized first around intervention design, followed by future research, and concluding with broader advocacy and policy considerations, these recommendations reflect the multiple domains in which action is needed to advance progress toward the goals articulated in Healthy People 2030. In Table 2, we further tailor these recommendations to the four intervention modalities examined in this review.

**TABLE 2:** Recommendations for advancing the effectiveness of lifestyle interventions on physical activity, cognitive and social engagement, diet/nutrition, and health monitoring to promote equity in ADRD outcomes.

Recommendation	Physical activity	Cognitive/social engagement	Diet/nutrition	Management of cardiovascular & metabolic risk factors
**1) Ongoing assessment of S/SDOH, including participant literacy and self‐reported experiences of stress**	Monitor changing factors like neighborhood conditions, transportation, or caregiving duties; adapt session timing or adjust format; link to culturally concordant stress management resources	Adjust training schedules and delivery based on participant life changes or barriers; link to culturally concordant stress management resources	Adapt dietary guidance based on employment status, food access, transportation, or household changes; link to culturally concordant stress management resources	Modify risk factor management support based on employment, caregiving responsibilities, or access to care; link to culturally concordant stress management resources
**2) Integration of culturally and contextually appropriate structural support**	Provide transportation or home‐based exercise options; address safety concerns; provide flexible scheduling	Offer culturally relevant cognitive tasks; provide technical support and community‐based sessions	Provide culturally relevant foods, meal stipends, or delivery; incorporate traditional cooking	Facilitate access to medications and flexible appointments; offer caregiver accommodations
**3) Integration of stakeholders/communities**	Co‐design exercise programs with community input to ensure relevance and cultural fit	Involve community in developing cognitive training content and delivery methods	Collaborate with local community organizations to include traditional diets and food practices	Engage community advisory boards to tailor cardiovascular/metabolic health education and care plans
**4) Integration of emerging biomarkers and data sources**	Add wearable activity, sleep, and mobility metrics to assess how structural conditions shape exercise engagement and response	Integrate digital cognitive performance measures, passive sensing of routine disruption, and neuroimaging or other biomarker data where feasible to assess how social and structural conditions influence cognitive intervention uptake, adherence, and effectiveness	Pair dietary intervention data with geocoded neighborhood food environment measures, air pollution or heat exposure estimates, and metabolomic or inflammatory biomarkers to capture how these factors shape dietary behavior	Link outcomes to physiologic biomarkers and access‐to‐care measures to better assess structural effects on risk factor control
**5) Advocacy and activism**	Partner with organizations to improve walkability and safe spaces; address zoning laws affecting exercise options	Advocate for inclusive educational resources and funding for cognitive health	Support policies reducing food deserts; advocate for affordable healthy foods, partnership with non‐profits like Meals on Wheels	Engage in policy change for improved eldercare infrastructure, access to health care, and health equity initiatives
**6) Considerations for diverse global contexts**	Adapt physical activity interventions considering pollution, safety, and cultural norms in diverse settings	Modify cognitive training for local cultural contexts; consider technology access globally	Adapt dietary interventions based on local foods, availability, and cultural preferences	Address local health‐care infrastructure and policy environments impacting risk factor management

Abbreviations: ADRD, Alzheimer's disease and related dementias; S/SDOH, social and structural determinants of health.

We first look at ongoing assessment of S/SDOH. ADRD prevention interventions should integrate ongoing, multilevel assessment of S/SDOH, including the evaluation of psychosocial stress. Rather than treating these determinants as static background variables, they should be actively monitored throughout implementation and used to guide adaptive strategies. For example, changes in neighborhood conditions, caregiving responsibilities, exposure to discrimination, or employment status could significantly influence adherence and should prompt modifications to intervention delivery as well as identification and linkage to appropriate stress‐mitigating resources. Formal assessment of participant literacy and health literacy should become standard practice in both trial enrollment and evaluation. This includes not only written literacy, but also digital, cultural, and health‐system literacy. Stratifying intervention effectiveness by literacy levels can reveal hidden disparities in comprehension, engagement, and outcomes, allowing for the tailoring of materials and delivery formats. Visual aids, plain‐language summaries, multilingual options, and oral communication channels may improve accessibility across diverse populations.

Integration of culturally appropriate structural support is the second recommendation. Structural supports must be embedded into program design—not as optional add‐ons, but as integral features that facilitate participation among marginalized groups. These supports should be tailored to the unique social contexts in which these groups are situated given that such contexts are shaped by interlocking multilevel forces that differently constrain or promote opportunities for health across differently socially positioned groups. Such supports include transportation assistance, childcare or caregiver accommodations, flexible scheduling, meal stipends or food delivery for dietary components, and digital access tools for technology‐based activities. Moreover, study design must not inherently exclude already marginalized populations. Providing these supports at scale can increase inclusivity, reduce attrition, and improve sustainability of interventions over time.

The third recommendation involves integration of stakeholders/communities as equal partners in research design and intervention. Integrating community members as equal partners in research design and implementation can be achieved by shifting from a top‐down approach toward collaboration. Strategies can include (1) workshops inviting community members to co‐design interventions and identify salient outcome measures for that community, as well as materials that reflect their lived experiences and cultural contexts; (2) community advisory boards, in which local representatives and advocates help guide research priorities, ethical implementation and dissemination plans; or (3) shared decision‐making frameworks and hiring community members onto the research team to promote equitable power sharing throughout the study. These approaches can ground research in mutual respect, relevance for the community, and sustainable impact.

Integration of emerging biomarkers and data sources is the fourth recommendation. Future ADRD intervention research should more explicitly integrate S/SDOH with emerging biological, behavioral, and environmental endpoints, including biomarkers and other novel measures that may better capture pathways through which structural inequities shape intervention effectiveness. Such measures may include inflammatory markers, epigenetic markers such as DNA methylation signatures, neuroimaging indicators, metabolomic and proteomic profiles, wearable‐derived activity and sleep metrics, passive sensing of mobility and routine disruption, air pollution and heat exposure estimates, and geocoded neighborhood deprivation indices. As ADRD science increasingly incorporates multidimensional data sources, it will be important to develop flexible frameworks that can accommodate new mechanisms, evolving exposure measures, and changing patterns of risk over time. Such an approach would strengthen the field's ability to identify how macro‐level conditions and micro‐level experiences jointly influence intervention response, while also improving the relevance of trials for diverse populations. Linking S/SDOH to emerging biomarkers and data infrastructures may ultimately support more precise, equitable, and contextually responsive ADRD prevention and intervention strategies.

Our fifth recommendation centers around advocacy and activism. Increasing avenues for community advocacy and macro‐level policy engagement is essential to address the upstream determinants that shape the feasibility of individual behavior change. While behavioral interventions can offer meaningful benefits, their impact will remain constrained without concurrent efforts to dismantle systemic barriers—such as discriminatory zoning laws, inadequate eldercare infrastructure, and underfunded public health systems. Building partnerships with advocacy organizations, investing in community‐based participatory research, and aligning with health equity coalitions can extend the reach of ADRD lifestyle interventions beyond the clinic or trial site and into the social and political environments that ultimately determine health.

Finally, we must consider diverse global contexts. One of the many benefits of the POINTER study is its global collaboration across multiple countries. Several of the included interventions have been used in multiple countries (see https://www.alz.org/wwfingers/overview.asp for more information). Therefore, researchers have an opportunity to investigate similar macro‐ and microstructure frameworks that are most relevant for their countries. For example, low‐ and middle‐income countries produce increasing proportions of the world's pollution and experience > 90% of the world's pollution‐related mortality as high‐income countries offload their pollution footprint elsewhere.^245^ Therefore, interrogating how pollution affects intervention implementation across the globe may provide valuable insights that can bolster global policy change. As political and economic systems become increasingly globalized, further attention should turn toward geopolitical and international alliances that can have influences on health policies in emerging economies.^246^ Additional studies^247^, ^248^, ^249^, ^250^, ^251^, ^252^, ^253^, ^254^, ^255^, ^256^, ^257^, ^258^, ^259^, ^260^, ^261^, ^262^, ^263^, ^264^, ^265^, ^266^, ^267^, ^268^, ^269^, ^270^, ^271^, ^272^, ^273^, ^274^, ^275^, ^276^, ^277^, ^278^, ^279^, ^280^, ^281^, ^282^, ^283^, ^284^, ^285^, ^286^, ^287^, ^288^, ^289^, ^290^, ^291^, ^292^, ^293^, ^294^, ^295^, ^296^, ^297^, ^298^, ^299^, ^300^, ^301^, ^302^, ^303^ to support these recommendations are included in Table 1.

## 5 CONCLUSIONS

To advance equitable outcomes in ADRD prevention, we must fundamentally reimagine how interventions are designed, implemented, and evaluated—moving beyond individual‐level frameworks to account for the multilevel social and structural forces that shape health behavior, health‐care access, and health outcomes. As our findings highlight, systemic inequities in resource access, stress exposure, and residential environment continue to undermine even the most promising interventions. Addressing these challenges requires not only culturally and contextually responsive programming, but also structural support, community‐driven research models, and policy engagement that center the lived realities of marginalized populations. Several studies—both observational and experimental—have demonstrated that inclusive and representative recruitment is possible, but without sustained attention to the social and structural conditions that determine who can engage, and who benefits, intervention equity will remain elusive. Future efforts must integrate critical theories, community expertise, and global collaboration to ensure ADRD interventions are not just evidence based, but justice oriented. Only by embedding equity into every level of intervention design and delivery can we ensure that brain health is a right, not a privilege.

## CONFLICT OF INTEREST STATEMENT

Bey: none to declare. Emrani: none to declare. Kulick: none to declare. Román: consultant for Cogstate, Steering; committee member for Accelerated Cure Project for Multiple Sclerosis (compensated), trustee for the Southern California Chapter of the National Multiple Sclerosis Society. De Anda‐Duran: none to declare. Ashford: none to declare. Boyle: none to declare. Bubu: none to declare. Dixon: none to declare. Franzen: served as a consultant to Biogen in 2022 (paid to her organization). Gao: none to declare. George: none to declare. Guest: none to declare. Heston: none to declare. Heyn: none to declare. Jackson: none to declare. Krishnamurthy: none to declare. Phillips: none to declare. Rirash: none to declare. Viswanathan: none to declare. Zanwar: associate editor, *Alzheimer's & Dementia: Behavior & Socioeconomics of Aging*. Zuelsdorff: none to declare. Stites: none to declare. Author disclosures are available in the Supporting Information.

## Supporting information



Supporting information: alz71957‐sup‐0001‐SuppMat.docx

Supporting information: alz71957‐sup‐0002‐Disclosureforms.pdf
