# Supplementary material for: Lifestyle interventions on Alzheimer's disease and related dementias: Social and structural determinants of implementation effectiveness and sustainability
Source: Alzheimers Dement. 2026 Nov 10;22(11):e71957. doi: 10.1002/alz.71957 (PMC13751840; doi:10.1002/alz.71957)
Supplement: Supplementary file 2 [file ALZ-22-e71957-s002.docx]

**Poverty and Income**

While poverty and income are often examined as a population-level risk factor for ADRD^1–5^, the more immediate effects on individuals’ capacity to participate in and adhere to lifestyle interventions remain underexplored. As previously described, participants living in poverty may face competing financial and survival priorities, such as food insecurity, unstable housing, inflexible work schedules, and transportation limitations, that undermine their ability to engage consistently with interventions. Poverty also co-occurs with psychosocial stressors that reduce the cognitive bandwidth and capacity to prioritize health behavior change^4^, ultimately increasing risk for future cognitive impairment. This creates a paradox: those who may benefit most from preventive interventions, due to heightened risk for ADRD from social disadvantage, are often those least able to engage with and sustain them.

Evidence from non-ADRD behavior change interventions focused on low-income groups found that they can be effective, especially those with fewer techniques and in the long-term for diet-related interventions^6,7^, but the evidence base is limited and inconsistent, with some studies showing poorer outcomes for lower-income participants. Differences in effectiveness may reflect pre-existing disparities in health behaviors rather than the interventions themselves.^7^ Moreover, community-level poverty is a key driver of neighborhood barriers to health-enabling infrastructure such as safe walking environments, health-promoting community centers or broadband internet access for digital components like home-based cognitive training.

*Knowledge gap:* Income is often underreported or unanalyzed in trials, and common measures (e.g., household income) may fail to capture relevant economic constraints, like area of residence, access to health clinics serving disadvantaged groups (e.g., federally qualified health centers), and medical insurance, thus remaining a major gap and area of future research.^8^ Further, inconsistent findings on whether participants with low income derive similar benefits from lifestyle interventions once economic barriers are addressed is an area that warrants further exploration.

*Recommendations:* Better measurements to capture aspects of income and poverty are needed. Intervention designs that recognize that health behaviors are constrained by economic hardship will allow for a better analysis of these structural conditions for more equitable impact. Further, ADRD lifestyle interventions that include mechanisms to offset the real-time impacts of poverty, as providing education to women and girls, economic opportunity in the workforce, transportation vouchers, digital devices and broadband support, child or elder care support, or culturally relevant food subsidies, can improve implementation and adherence.

**Physical activity**

Trials have strengthened the case for exercise-anchored dementia-risk reduction, but they also reveal a trade-off: the most effective approaches often require resources (customization, coaches, supervision, social support transportation, program fees) that can constrain scalability, access, and inclusion.^9–12^ Both macrosystem factors (e.g., cultural norms and legal/religious factors that restrict participation) and microsystem factors (e.g., neighborhood safety, built environment, availability of peers, transportation and personal health status) may influence people’s ability to engage in or benefit from physical activity.^9,13^ Mobility limitations, multimorbidity, sensory changes, and fear of falling are important factors when considering exercise prescription for brain health.^14,15^ These constraints require individualized modality prescriptions that prioritize functional ability, safety (e.g., supervised, progressive resistance; chair-based or aquatic options; assistive device integration; home hazard mitigation), realistic dosing, and monitoring.^16,17^

As we have described in this review, Healthy People 2030 identified five key S/SDOH domains that shape a person’s ability to initiate and sustain physical activity and should be considered for future intervention studies. Early work in Federally Qualified Health Centers (FQHCs) shows that physical activity referral programs are adoptable in underserved settings when clinics can offer on-site or nearby options and address transport and cost barriers.^18,19^ Other implementation guides exist including structured exercise prescriptions, enlisting qualified community programs, and standardized SDOH screening to determine needs for those engaging in physical activity.^20,21^ Other resources, including community care-referral platforms such as Unite Us and Findhelp, and payer supports (e.g., Medicare Advantage fitness and transportation supplemental benefits) can be incorporated to reduce out-of-pocket burden.^22–24^

*Knowledge gap:* Physical activity is one of the most well-studied lifestyle interventions in ADRD research. Yet, few trials have examined how important resources and determinants of engagement in physical activity, including neighborhood safety, mobility, multimorbidity, cultural norms, transportation access, and other micro-level factors intersect to influence the uptake and sustainability of interventions on physical activity across diverse populations.

*Recommendations:* Treating exercise not only as medicine, but as a socially determined health behavior that must be prescribed and resourced within the broader system of care could help bolster equitable implementation. ^77^ Future intervention studies may consider building clinic-to-community networks and funding transport and adaptive options, standardizing benefits for exercise programs including rides/transportation for older adults with functional limitations^15,25^ and providing age-appropriate activities for older adults and ensuring opportunities for social interaction and support.^26,27^

**Education**

Educational attainment is a key microsystem-level determinant influencing the implementation and efficacy of interventions targeting ADRD. Extensive evidence underscores the protective influence of education against cognitive decline.^15^ Moreover, ongoing health education is recognized as a critical component within ADRD interventions, facilitating cognitive health through targeted knowledge dissemination, skill-building, and empowerment strategies that promote sustained behavioral changes across diverse populations.^28^

While the evidence base is smaller, education has been shown to also notably influence health-behavior intervention adherence by improving health literacy, enhancing self-efficacy, and increasing resource accessibility.^29,30^ However, these benefits vary significantly by socioeconomic and racial groups, with higher-educated individuals generally demonstrating greater engagement in preventive behaviors and cognitive training and therefore deriving greater cognitive benefits.^31^ Encouragingly, culturally adapted community-based health education initiatives have successfully increased dementia-preventive behaviors among racially minoritized populations, highlighting the potential to address educational disparities through tailored interventions.^28^

*Knowledge gap:* Many studies measure education as years of schooling or degree attainment, which do not always reflect educational quality. Studies should investigate how comprehensive questionnaires that extend beyond binned educational attainment (e.g., some high school/high school etc.) better characterize the effects of education on ADRD lifestyle intervention efficacy.

*Recommendations:* Intervention materials should emphasize empowerment, skill-building, and practical application. Future research and policy initiatives must prioritize educational equity and culturally sensitive intervention strategies to enhance the effectiveness and sustainability of ADRD prevention efforts across diverse populations.

1.	Strashun A, Ortega C, Horn L, MAGAi C. THE EFFECTS OF POVERTY AND EDUCATION ON TEMPOROPARIETAL PERFUSION IN ALZHEIMER’S DISEASE: A RECONSIDERATION OF THE CEREBRAL RESERVE HYPOTHESIS. *Int. J. Geriatr. Psychiatry* [Internet]. 1996 [cited 2026 Mar 16];11:105–106. Available from: /doi/pdf/10.1002/%28SICI%291099-1166%28199612%2911%3A12%3C1105%3A%3AAID-GPS430%3E3.0.CO%3B2-7

2.	Maalouf M, Fearon M, Lipa MC, Chow- Johnson H, Tayeh L, Lipa D. Neurologic Complications of Poverty: the Associations Between Poverty as a Social Determinant of Health and Adverse Neurologic Outcomes. *Curr. Neurol. Neurosci. Rep.* [Internet]. 2021 [cited 2026 Mar 16];21. Available from: https://pubmed.ncbi.nlm.nih.gov/33948738/

3.	Hyun J, Schiff M, Hall CB, Appelhans BM, Barinas-Mitchell E, Thurston RC, Karvonen-Gutierrez CA, Hedderson MM, Janssen I, Derby CA. Exposure to neighborhood concentrated poverty is associated with faster decline in episodic memory among midlife women. *Alzheimers Dement.* [Internet]. 2025 [cited 2026 Mar 16];21. Available from: https://pubmed.ncbi.nlm.nih.gov/40189810/

4.	Appelhans BM. The Cognitive Burden of Poverty: a Mechanism of Socioeconomic Health Disparities. *Am. J. Prev. Med.* [Internet]. 2022 [cited 2026 Mar 16];64:293. Available from: https://pmc.ncbi.nlm.nih.gov/articles/PMC10176429/

5.	de Havenon A, Stulberg EL, Littig L, Wong KH, Sarpong D, Li V, Sharma R, Falcone GJ, Williamson JD, Pajewski NM, et al. Socioeconomic and medical determinants of state-level subjective cognitive decline in the United States. *Alzheimers Dement.* [Internet]. 2024 [cited 2026 Mar 16];20:7567–7579. Available from: https://pubmed.ncbi.nlm.nih.gov/39351858/

6.	McGill R, Anwar E, Orton L, Bromley H, Lloyd-Williams F, O’Flaherty M, Taylor-Robinson D, Guzman-Castillo M, Gillespie D, Moreira P, et al. Are interventions to promote healthy eating equally effective for all? Systematic review of socioeconomic inequalities in impact. *BMC Public Health* [Internet]. 2015 [cited 2025 Oct 6];15. Available from: https://pubmed.ncbi.nlm.nih.gov/25934496/

7.	Michie S, Jochelson K, Markham WA, Bridle C. Low-income groups and behaviour change interventions: a review of intervention content, effectiveness and theoretical frameworks. *J. Epidemiol. Community Health (1978).* [Internet]. 2009 [cited 2025 Oct 7];63:610–622. Available from: https://pubmed.ncbi.nlm.nih.gov/19386612/

8.	Bull ER, Dombrowski SU, McCleary N, Johnston M. Are interventions for low-income groups effective in changing healthy eating, physical activity and smoking behaviours? A systematic review and meta-analysis. *BMJ Open* [Internet]. 2014 [cited 2025 Oct 7];4. Available from: https://pubmed.ncbi.nlm.nih.gov/25432903/

9.	Baker LD, Espeland MA, Whitmer RA, Snyder HM, Leng X, Lovato L, Papp K V., Yu M, Kivipelto M, Alexander AS, et al. Structured vs Self-Guided Multidomain Lifestyle Interventions for Global Cognitive Function: The US POINTER Randomized Clinical Trial. *JAMA* [Internet]. 2025 [cited 2025 Oct 6];334:681–691. Available from: https://jamanetwork.com/journals/jama/fullarticle/2837046

10.	Ngandu T, Lehtisalo J, Solomon A, Levälahti E, Ahtiluoto S, Antikainen R, Bäckman L, Hänninen T, Jula A, Laatikainen T, et al. A 2 year multidomain intervention of diet, exercise, cognitive training, and vascular risk monitoring versus control to prevent cognitive decline in at-risk elderly people (FINGER): a randomised controlled trial. *The Lancet* [Internet]. 2015 [cited 2025 Oct 6];385:2255–2263. Available from: https://www.sciencedirect.com/science/article/pii/S0140673615604615

11.	Sääskilahti M, Kulmala J, Nurhonen M, Lehtisalo J, Peltonen M, Mangialasche F, Laatikainen T, Strandberg T, Antikainen R, Tuomilehto J, et al. The effect of multidomain lifestyle intervention on health care service use and costs - secondary analyses from the Finnish Geriatric Intervention Study to Prevent Cognitive Impairment and Disability (FINGER): a randomised controlled trial. *Age Ageing* [Internet]. 2024 [cited 2025 Oct 6];53:afae249. Available from: https://pmc.ncbi.nlm.nih.gov/articles/PMC11584201/

12.	Deckers K, Zwan MD, Soons LM, Waterink L, Beers S, van Houdt S, Stiensma B, Kwant JZ, Wimmers SCPM, Heutz RAM, et al. A multidomain lifestyle intervention to maintain optimal cognitive functioning in Dutch older adults-study design and baseline characteristics of the FINGER-NL randomized controlled trial. *Alzheimers Res. Ther.* [Internet]. 2024 [cited 2025 Oct 6];16. Available from: https://pubmed.ncbi.nlm.nih.gov/38872204/

13.	Barnett DW, Barnett A, Nathan A, Van Cauwenberg J, Cerin E. Built environmental correlates of older adults’ total physical activity and walking: a systematic review and meta-analysis. *Int. J. Behav. Nutr. Phys. Act.* [Internet]. 2017 [cited 2025 Oct 6];14. Available from: https://pubmed.ncbi.nlm.nih.gov/28784183/

14.	Maresova P, Krejcar O, Maskuriy R, Bakar NAA, Selamat A, Truhlarova Z, Horak J, Joukl M, Vítkova L. Challenges and opportunity in mobility among older adults – key determinant identification. *BMC Geriatr.* [Internet]. 2023 [cited 2025 Oct 6];23:1–29. Available from: https://bmcgeriatr.biomedcentral.com/articles/10.1186/s12877-023-04106-7

15.	Livingston G, Huntley J, Liu KY, Costafreda SG, Selbæk G, Alladi S, Ames D, Banerjee S, Burns A, Brayne C, et al. Dementia prevention, intervention, and care: 2024 report of the Lancet standing Commission. *The Lancet* [Internet]. 2024 [cited 2025 Jun 8];404:572–628. Available from: https://pubmed.ncbi.nlm.nih.gov/39096926/

16.	Dayao JKO, Duffy CEL, Cristiano AM, Kallenberg GR, Linke SE. Implementation and evaluation of Exercise is Medicine in primary care clinics within a large academic health system. *Fam. Med. Community Health* [Internet]. 2024 [cited 2025 Oct 6];12. Available from: https://pubmed.ncbi.nlm.nih.gov/38307706/

17.	Nicholson WK, Silverstein M, Wong JB, Barry MJ, Chelmow D, Coker TR, Davis EM, Jaén CR, Krousel-Wood M, Lee S, et al. Interventions to Prevent Falls in Community-Dwelling Older Adults: US Preventive Services Task Force Recommendation Statement. *JAMA* [Internet]. 2024 [cited 2025 Oct 6];332:51–57. Available from: https://pubmed.ncbi.nlm.nih.gov/38833246/

18.	Ylitalo KR, Cox W, Gutierrez M, Benavidez G, Umstattd Meyer MR, Niceler B, Griggs JO. A Prescription for Wellness: Exercise Referrals at a Federally Qualified Health Center. *J. Prim. Care Community Health* [Internet]. 2020 [cited 2025 Oct 6];11. Available from: https://pubmed.ncbi.nlm.nih.gov/32674714/

19.	Office of the Assistant Secretary for Planning and Evaluation (ASPE) at the U.S. Department of Health & Human Services. Social Determinants of Health Data Sharing at the Community Level. 2021;

20.	Lin CY, Gentile NL, Bale L, Rice M, Lee ES, Ray LS, Ciol MA. Implementation of a Physical Activity Vital Sign in Primary Care: Associations Between Physical Activity, Demographic Characteristics, and Chronic Disease Burden. *Prev. Chronic Dis.* [Internet]. 2022 [cited 2025 Oct 6];19. Available from: https://pubmed.ncbi.nlm.nih.gov/35749145/

21.	Welcome to the Physical Activity IG - Physical Activity Implementation Guide v1.0.1 [Internet]. [cited 2025 Oct 6];Available from: https://build.fhir.org/ig/HL7/physical-activity/

22.	Message from Unite Us [Internet]. [cited 2025 Oct 6];Available from: https://uniteus.com/products/closed-loop-referral-system/

23.	Center for Health Care Strategies | Better care where it’s needed most [Internet]. [cited 2026 Mar 16];Available from: https://www.chcs.org/

24.	Breed Mayor L. SAN FRANCISCO BEHAVIORAL HEALTH SERVICES DRUG MEDI-CAL ORGANIZED DELIVERY SYSTEM QUALITY IMPROVEMENT WORK PLAN EVALUATION REPORT FY 2022-2023 City and County of San Francisco DEPARTMENT OF PUBLIC HEALTH Quality Improvement Work Plan Evaluation Report FY ….

25.	Shen Y, Hu X, Nipp RD, Yabroff KR, Hong AS, Liao JM, Jiang C. Nonemergency Medical Transportation Benefit in Traditional Medicare Advantage and Value-Based Plans. *JAMA Netw. Open* [Internet]. 2024 [cited 2025 Oct 6];7:e2449038. Available from: https://pmc.ncbi.nlm.nih.gov/articles/PMC11618458/

26.	West ST, Wu X, Liechty T, Son JS, Juris J, Janke M, Wong J, Chen G. Today’s Older Adults: Strategies of Facilitating Sport Participation. *J. Park Recreat. Admi.* [Internet]. 2024 [cited 2025 Oct 6];42:41–60. Available from: https://experts.illinois.edu/en/publications/todays-older-adults-strategies-of-facilitating-sport-participatio

27.	Jenkin CR, Eime RM, van Uffelen JGZ, Westerbeek H. How to re-engage older adults in community sport? Reasons for drop-out and re-engagement. *Leisure Studies* [Internet]. 2021 [cited 2025 Oct 6];40:441–453. Available from: https://www.tandfonline.com/doi/pdf/10.1080/02614367.2021.1888310

28.	Chandler MJ, Locke DE, Crook JE, Fields JA, Ball CT, Phatak VS, Dean PM, Morris M, Smith GE. Comparative Effectiveness of Behavioral Interventions on Quality of Life for Older Adults With Mild Cognitive Impairment: A Randomized Clinical Trial. *JAMA Netw. Open* [Internet]. 2019 [cited 2025 Oct 6];2. Available from: https://pubmed.ncbi.nlm.nih.gov/31099860/

29.	Farley H. Promoting self-efficacy in patients with chronic disease beyond traditional education: A literature review. *Nurs. Open* [Internet]. 2019 [cited 2025 Oct 23];7:30–41. Available from: https://pubmed.ncbi.nlm.nih.gov/31871689/

30.	Miller TA. Health literacy and adherence to medical treatment in chronic and acute illness: A meta-analysis. *Patient Educ. Couns.* [Internet]. 2016 [cited 2025 Oct 6];99:1079–1086. Available from: https://www.sciencedirect.com/science/article/pii/S0738399116300416?via%3Dihub

31.	Pampel FC, Krueger PM, Denney JT. Socioeconomic Disparities in Health Behaviors. *Annu. Rev. Sociol.* [Internet]. 2010 [cited 2025 Oct 23];36:349–370. Available from: https://pubmed.ncbi.nlm.nih.gov/21909182/
